# Cell layer–specific expression of the homeotic MADS-box transcription factor PhDEF contributes to modular petal morphogenesis in petunia

**DOI:** 10.1093/plcell/koad258

**Published:** 2023-10-06

**Authors:** Mathilde Chopy, Quentin Cavallini-Speisser, Pierre Chambrier, Patrice Morel, Jérémy Just, Véronique Hugouvieux, Suzanne Rodrigues Bento, Chloe Zubieta, Michiel Vandenbussche, Marie Monniaux

**Affiliations:** Laboratoire de Reproduction et Développement des Plantes, Université de Lyon, ENS de Lyon, UCB Lyon 1, CNRS, INRAE, Lyon 69007, France; Laboratoire de Reproduction et Développement des Plantes, Université de Lyon, ENS de Lyon, UCB Lyon 1, CNRS, INRAE, Lyon 69007, France; Laboratoire de Reproduction et Développement des Plantes, Université de Lyon, ENS de Lyon, UCB Lyon 1, CNRS, INRAE, Lyon 69007, France; Laboratoire de Reproduction et Développement des Plantes, Université de Lyon, ENS de Lyon, UCB Lyon 1, CNRS, INRAE, Lyon 69007, France; Laboratoire de Reproduction et Développement des Plantes, Université de Lyon, ENS de Lyon, UCB Lyon 1, CNRS, INRAE, Lyon 69007, France; Laboratoire de Physiologie Cellulaire et Végétale, Université Grenoble-Alpes, CNRS, CEA, INRAE, IRIG-DBSCI, Grenoble 38000, France; Laboratoire de Reproduction et Développement des Plantes, Université de Lyon, ENS de Lyon, UCB Lyon 1, CNRS, INRAE, Lyon 69007, France; Laboratoire de Physiologie Cellulaire et Végétale, Université Grenoble-Alpes, CNRS, CEA, INRAE, IRIG-DBSCI, Grenoble 38000, France; Laboratoire de Reproduction et Développement des Plantes, Université de Lyon, ENS de Lyon, UCB Lyon 1, CNRS, INRAE, Lyon 69007, France; Laboratoire de Reproduction et Développement des Plantes, Université de Lyon, ENS de Lyon, UCB Lyon 1, CNRS, INRAE, Lyon 69007, France

## Abstract

Floral homeotic MADS-box transcription factors ensure the correct morphogenesis of floral organs, which are organized in different cell layers deriving from distinct meristematic layers. How cells from these distinct layers acquire their respective identities and coordinate their growth to ensure normal floral organ morphogenesis is unresolved. Here, we studied petunia (*Petunia* × *hybrida*) petals that form a limb and tube through congenital fusion. We identified petunia mutants (periclinal chimeras) expressing the B-class MADS-box gene *DEFICIENS* in the petal epidermis or in the petal mesophyll, called *wico* and *star*, respectively. Strikingly, *wico* flowers form a strongly reduced tube while their limbs are almost normal, while *star* flowers form a normal tube but greatly reduced and unpigmented limbs, showing that petunia petal morphogenesis is highly modular. These mutants highlight the layer-specific roles of PhDEF during petal development. We explored the link between PhDEF and petal pigmentation, a well-characterized limb epidermal trait. The anthocyanin biosynthesis pathway was strongly downregulated in *star* petals, including its major regulator *ANTHOCYANIN2* (*AN2*). We established that PhDEF directly binds to the *AN2* terminator in vitro and in vivo, suggesting that PhDEF might regulate *AN2* expression and therefore petal epidermis pigmentation. Altogether, we show that cell layer–specific homeotic activity in petunia petals differently impacts tube and limb development, revealing the relative importance of the different cell layers in the modular architecture of petunia petals.

## Teaser

The expression of B-class homeotic MADS-box transcription factor *PhDEF* in different cell layers drives petunia petal tube or limb development.

**IN A NUTSHELL**

**Background:** Petals are not only beautiful, but they are also very important floral organs that have coevolved with different animal visitors to ensure pollination. This long coevolution produced many complex petal shapes. In the case of *Petunia*, the fused petals are organized in 2 domains, the tube and the limb; this influences the interaction of the flower with hawk moths, hummingbirds, or bees. Petal identity genes, such as *PhDEFICIENS* (*PhDEF*), trigger petal development resulting in mature petals. However, the mechanisms by which those genes drive complex petal shape with tube and limb are unclear.

**Question:** Petals are formed of cell layers: the epidermis and the internal cells. In a wild-type flower, the petal identity gene *PhDEF* is expressed in all cell layers. But what happens if *PhDEF* expression is restricted to a specific cell layer? In other words, we wanted to investigate the layer-specific contribution of *PhDEF* in petal tube and limb development.

**Findings:** By chance, we obtained the perfect material to address this question: 2 categories of *Petunia hybrida* mutants (chimeras) expressing *PhDEF* exclusively in the petal epidermis or in the inner cells, called *wico* and *star*, respectively. The resulting flowers displayed dramatically different limb and tube shape (see picture): *wico* flowers form a strongly reduced tube while their limb is almost normal, and *star* flowers form a normal tube but a very reduced limb. This suggests that petunia petal morphogenesis is highly modular and depends on the cell layer–specific expression of *PhDEF*.

**Next steps:** This study is a first step toward understanding the link between *PhDEF* and complex petal development. A major future challenge is to identify the genes acting downstream of the petal identity genes, at the tissue (epidermis versus internal cells) and organ (limb versus tube) scales.

## Introduction

All plant aerial organs derive from clonally distinct layers, named L1, L2, and L3 in the shoot apical meristem (SAM) (Satina et al. 1940). Within the L1 and L2 layers, cells divide anticlinally, thereby maintaining a clear layered structure in all aerial organs produced by the SAM (Stewart and Burk 1970; Meyerowitz 1997; Scheres 2001). Already at the embryonic stage, meristematic cell layers express different genes and have distinct identities (Lu et al. 1996; Abe et al. 1999) that are maintained in the adult SAM (Yadav et al. 2014). During flower development, floral organ identity will be appended on top of layer identity by the combinatorial expression of homeotic floral genes, most of which are MADS-box genes (Schwarz-Sommer et al. 1990; Coen and Meyerowitz 1991). How these master floral regulators specify all floral organ features, such as organ size, shape, pigmentation, and cellular properties, while maintaining layer-specific identities, is unknown.

Petals are often the most conspicuous organs of the flower, and they display a tremendous diversity in size, shape, and pigmentation across flowering plants (Moyroud and Glover 2017). Floral organ identity is specified by a combination of A-, B- and C-class identity genes as proposed by the classical ABC model established in Arabidopsis (*Arabidopsis thaliana*) and snapdragon (*Antirrhinum majus*), and B-class genes are particularly important for petal identity (Schwarz-Sommer et al. 1990; Coen and Meyerowitz 1991; Morel et al. 2017). B-class proteins, belonging to MADS-box transcription factors (TFs), are grouped in the DEF/AP3 and the GLO/PI subfamilies, named after the snapdragon/Arabidopsis B-class proteins DEFICIENS/APETALA3 and GLOBOSA/PISTILLATA (Purugganan et al. 1995; Theißen et al. 1996). These proteins act as obligate heterodimers consisting of one DEF/AP3 and one GLO/PI protein, together with other MADS-box TFs of the SEPALLATA subfamily (Melzer et al. 2009), and this complex activates the expression of *DEF/AP3* and *GLO/PI* genes for maintenance of high expression levels throughout petal and stamen development (Tröbner et al. 1992).

In petunia (*Petunia* × *hybrida*, abbreviated *Ph* for gene names), gene duplication has generated 4 B-class genes, namely *PhDEF* (*DEFICIENS*) and *PhTM6* (*TOMATO MADS-BOX GENE6*) belonging to the *DEF/AP3* subfamily and *PhGLO1* (*GLOBOSA1*) and *PhGLO2* (*GLOBOSA2*) belonging to the *GLO/PI* subfamily (Angenent et al. 1992; van der Krol et al. 1993; Vandenbussche et al. 2004; Rijpkema et al. 2006). Mutating the 2 members of each subfamily (*phdef phtm6* or *phglo1 phglo2* double mutants) results in a classical B-function mutant phenotype with homeotic transformation of petals into sepals and stamens into carpels (Vandenbussche et al. 2004; Rijpkema et al. 2006). Additionally, gene copies within the *DEF/AP3* subfamily have diverged in function: while *PhDEF* exhibits a classical B-class expression pattern largely restricted to developing petals and stamens, *PhTM6* is atypically expressed in stamens and carpels, and its upregulation depends on the petunia C-function genes (Rijpkema et al. 2006; Heijmans et al. 2012). As a consequence, the single *phdef* mutant displays a homeotic conversion of petals into sepals, while the stamens are normal due to functional redundancy with *PhTM6* (Rijpkema et al. 2006). The petunia *phdef* mutant is therefore an interesting model to study the mechanism of petal identity specification alone since it displays a single-whorl complete homeotic transformation, which is quite rare for floral homeotic mutants that generally show defects in 2 adjacent whorls.

Flowers from the *Petunia* genus develop 5 petals that arise as individual primordia and fuse congenitally (Vandenbussche et al. 2009). Mature petals are fully fused, and the corolla is organized in 2 distinct domains: the tube and the limb. Variation in the relative size of the tube and the limb is observed among wild species of *Petunia*, where flowers with a long tube grant nectar access to long-tongued hawk moths or hummingbirds, while wide and short tubes are easily accessible to bees (Galliot et al. 2006). The short- and long-tube species cluster separately on a phylogeny of wild *Petunia* species, and the short-tube phenotype is likely the ancestral one (Reck-Kortmann et al. 2014). Pollinator preference assays and field observations have confirmed that tube length and limb size are discriminated by pollinators and thereby might play a role in reproductive isolation, together with multiple other traits of the pollination syndromes such as limb pigmentation or volatile emission (Galliot et al. 2006; Hoballah et al. 2007; Venail et al. 2010). Tube and limb therefore appear to act as different functional modules in the petunia flower.

Although the petunia petal tube and limb seem to play important ecological roles, the mechanisms driving their development are mostly unknown. Tube and limb develop as relatively independent entities in flowers from the Solanaceae family, to which petunia belongs: for instance, tube length and limb width are uncorrelated traits in intraspecific crosses performed in *Nicotiana* or *Jaltomata* (Bissell and Diggle 2008; Kostyun et al. 2019). Moreover, tube and limb identities can be acquired independently: this is strikingly observed in the petunia *blind* mutant, a partial A-class mutant that forms an almost wild-type (wt) tube topped by functional anthers, due to ectopic C-class activity in the second whorl (Cartolano et al. 2007). Apart from the petal identity genes, the molecular players involved in petunia tube or limb morphogenesis are mostly unknown. General growth factors affect petal development as a whole (both tube and limb) together with other vegetative or reproductive traits (Vandenbussche et al. 2009; Terry et al. 2019; Brandoli et al. 2020), but very few genes have been found to specifically affect growth of one subdomain of the petal (Zenoni et al. 2004). Therefore, the mechanisms of petunia tube and limb morphogenesis remain to be fully explored.

In contrast, the genetic and molecular bases of petunia petal pigmentation are extremely well characterized, thanks to the plethora of mutants that have been isolated over decades of breeding and research (Tornielli et al. 2009; Bombarely et al. 2016). Petunia limb pigmentation is mainly due to the accumulation of anthocyanins in the vacuole of adaxial epidermal cells. Briefly, the earliest steps of anthocyanin production are ensured by a MBW regulatory complex composed of an R2R3-*M*YB TF (either ANTHOCYANIN2 [AN2], AN4, DEEP PURPLE [DPL], or PURPLE HAZE), a *b*HLH TF (AN1 or JAF13), and a *W*D-40 repeat protein (AN11), which drives the expression of anthocyanin biosynthesis enzymes and proteins involved in vacuolar acidification of epidermal cells (Quattrocchio et al. 1993, 1998, 1999; de Vetten et al. 1997; Spelt et al. 2000; Albert et al. 2011). How this pathway is activated, after regulators such as PhDEF have specified petal identity, has not been elucidated so far.

In this work, we present petunia flowers with strongly affected tube or limb development, which we respectively named *wico* and *star*, and that spontaneously arose from *phdef-151* mutant plants. We provide genetic and molecular evidence that both of these flower types are periclinal chimeras, resulting from the layer-specific excision of the transposon inserted into the *PhDEF* gene, restoring *PhDEF* activity either in the epidermis or in the mesophyll of the petal. The *star* and *wico* phenotypes indicate that in the petunia petal, the epidermis mainly drives limb morphogenesis while the mesophyll mainly drives tube morphogenesis. This is seemingly different from previous studies in snapdragon flowers, another species with fused petals, where *def* periclinal chimeras indicated that epidermal *DEF* expression was making a major contribution to overall petal morphology (Perbal et al. 1996; Efremova et al. 2001; Vincent et al. 2003). We characterized in detail the *star* and *wico* petal phenotypes at the tissue and cellular scale and found evidence for non-cell-autonomous effects affecting cell identity between layers. We sequenced the total petal transcriptome from wt, *wico*, and *star* flowers at 3 developmental stages, and we found that a large proportion of the genes involved in anthocyanin production were downregulated in *star* petal samples, as could be expected from their white petals. We further showed, by gel shift assay and chromatin immunoprecipitation (ChIP), that PhDEF binds to the terminator region of *AN2*, thereby possibly regulating its expression and triggering the first steps of limb pigmentation. Our results and our unique flower material promise to improve our understanding of tube and limb morphogenesis in petunia and address the broader question of how organ identity and cell layer identity overlap during organ development.

## Results

### Spontaneous appearance of 2 phenotypically distinct classes of partial revertants from the *phdef-151* locus

**Figure 1.**
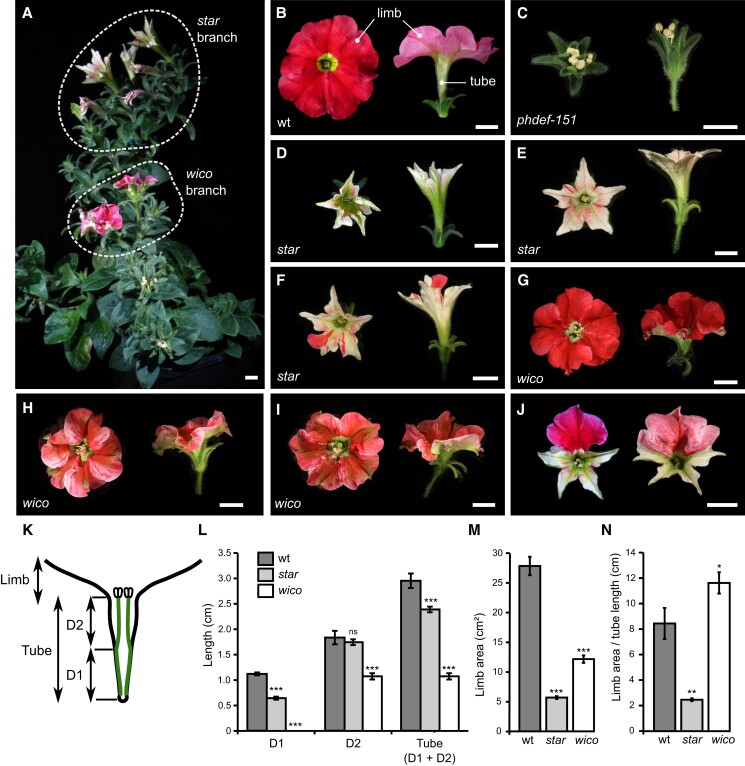
Macroscopic description of the *star* and *wico* flowers. **A)***phdef-151* mutant plant harboring 1 branch with *wico* revertant flowers and 1 branch with *star* revertant flowers. Scale bar: 1 cm. **B to I)** Representative wt **B)**, *phdef-151***C)**, *star***D to F)**, and *wico***G to I)** flowers from a top (left) and side (right) view. The *star* and *wico* flowers come from independent reversion events (from different *phdef-151* plants or from different branches of a single *phdef-151* plant). Scale bar: 1 cm. **J)** Two *star* flowers with additional L1-revertant sectors in 1 petal (left) or 1 petal and 2 half petals (right). Scale bar: 1 cm. **K)** Schematic cross-section of a wt flower, showing stamens (in green and with anthers depicted at the top) partially fused to the petal tube. The region of the tube fused to stamens is named D1, and the region of the tube where stamens are free is named D2, as defined in Stuurman et al. (2004). **L)** Average length of regions D1 and D2 and total tube length in wt, *star*, and *wico* flowers. **M)** Average limb area in wt, *star*, and *wico* flowers. **N)** Average ratio between limb area and tube length in wt, *star*, and *wico* flowers. *n* = 7 wt flowers, *n* = 12 *star* flowers from 4 different branches, and *n* = 18 *wico* flowers from 5 different branches. Student's *t* test, two-sided with Welch correction for D1, D2, and tube length and two-sided without Welch correction for limb area and limb area/tube length ratio (*P < 0.05, **P < 0.01, ***P < 0.005; *ns*, non-significant *P* > 0.05). Error bars represent ±SEM.

Previously described null alleles for the *PhDEF* gene (also named *GP* or *pMADS1*) were obtained by either EMS mutagenesis (de Vlaming et al. 1984; Rijpkema et al. 2006) or by γ-radiation (van der Krol et al. 1993). Because neither of these alleles was straightforward to genotype in a heterozygous state, we screened our sequence-indexed *dTph1* transposon mutant population in the W138 genetic background (Vandenbussche et al. 2008) for other insertions into *PhDEF*. We identified a mutant allele named *phdef-151*, referring to the *dTph1* insertion 151 bp downstream of the ATG in the first exon of the *PhDEF* gene, predicted to fully disrupt the MADS domain in the protein sequence by premature termination of the first exon due to multiple stop codons in the different reading frames of *dTph1*. As observed for previously identified *phdef* null alleles, *phdef-151* flowers display a complete homeotic conversion of petals into sepals, while heterozygous or homozygous wt siblings display red-colored wt petals (Fig. 1, A to C). *phdef-151* is thus very likely a null mutant allele.

While growing homozygous *phdef-151* individuals during several seasons, we repeatedly observed the spontaneous appearance of inflorescence side branches that developed flowers with a partial restoration of petal development (Fig. 1; Supplemental Fig. S1), suggesting excision of the *dTph1* transposon from the *phdef-151* allele specifically in these side branches. Remarkably, these partially revertant flowers could be classified as belonging to either 1 of 2 contrasting phenotypic classes, which we named *star* and *wico*, and that could even occur simultaneously in different branches on the same plant (Fig. 1A). For both phenotypic classes, we obtained more than 15 independent reversion events. The *star* flowers (Fig. 1, D to F), named in reference to their star-shaped petals, grow an elongated tube similar to wt flowers, but their limbs are underdeveloped: they appear to mainly grow around the midvein with strongly reduced lateral expansion, hence losing the typical round shape of wt limb. Moreover, they have almost white petals, suggesting strongly reduced accumulation of anthocyanins.

We quantified the changes in flower morphology (Fig. 1, K to N) and found that total limb area was reduced almost 5-fold in *star* flowers (Fig. 1M). In contrast, total tube length was only slightly reduced (by 19%) in *star* as compared to wt (Fig. 1L), and this was mainly due to a reduction in length of domain D1, corresponding to the part of the tube fused with stamens (as defined in Stuurman et al. 2004; Fig. 1K), while length of the rest of the tube (domain D2) remained unchanged (Fig. 1L; Supplemental Fig. S2). As a result, the ratio between limb area and tube length, which we use as a simple measure for overall corolla morphology, is reduced about 4-fold in *star* flowers as compared to wt (Fig. 1N). In addition, we occasionally observed fully pigmented secondary revertant sectors of various sizes in the *star* genetic background, in some cases leading to the development of a single wt-like petal in a *star* flower background (Fig. 1J). These revertant sectors, observed multiple times, always exhibited simultaneous restoration of pigmentation and normal petal limb growth patterns, demonstrating that the strongly reduced pigmentation in *star* petals was due to impaired PhDEF function and not to an additional mutation in the pigmentation pathway.

The *wico* flowers, named after their *wi*de *co*rolla, grow round-shaped and pigmented limbs while their tube remains underdeveloped (Fig. 1, G to I). Limb pigmentation ranged from pink to bright red, and green sepaloid tissue was observed around the midveins, commonly well visible in all *wico* flowers on the abaxial side of the petals (see for instance Supplemental Fig. S1E). Total tube length was reduced about 3-fold in *wico* flowers, with domain D1 being absent since stamens were totally unfused to the tube (Supplemental Fig. S2), while domain D2 was significantly reduced in size compared to wt (Fig. 1L). Limb area was also about 2-fold reduced in *wico* as compared to wt flowers (Fig. 1M), but the ratio between limb area and tube length was higher than in wt flowers (Fig. 1N), indicating the larger contribution of limb tissue to total corolla morphology in *wico* flowers. In summary, the *star* flowers form an almost normal tube but small, misshaped, and unpigmented limbs, while the *wico* flowers form almost normally shaped and pigmented limbs but a tube strongly reduced in length. These contrasting phenotypes suggest that tube and limb development can be uncoupled in petunia flowers, at least to some degree.

### The *star* and *wico* flowers result from excision of the *dTph1* transposon from the *phdef-151* locus

Reversion of a mutant phenotype toward a partial or a complete wt phenotype is classically observed in unstable transposon insertion mutant alleles. In the petunia W138 line from which *phdef-151* originates, the *dTph1* transposon is actively transposing (Gerats et al. 1990). We assumed therefore that the *star* and *wico* flowers were caused by the excision of *dTph1* from the *PhDEF* locus. *dTph1* transposition is generally accompanied by an 8-bp duplication of the target site upon insertion, and excision can have various outcomes depending on the length and nature of the remaining footprint (van Houwelingen et al. 1999). Hence, we first hypothesized that the distinct *star* and *wico* phenotypes were caused by different types of alterations of the *PhDEF* coding sequence after the excision of *dTph1*.

**Figure 2.**
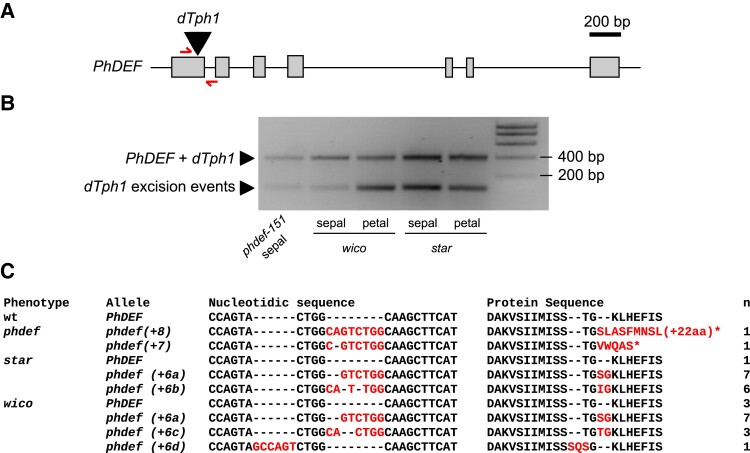
Sequencing the *PhDEF* excision alleles in *star* and *wico* flowers. **A)***PhDEF* gene model indicating the position of the *dTph1* insertion in the first exon (black triangle) and the primers used for subsequent amplification and sequencing (red half arrows). **B)** Amplicons generated with primers spanning the *dTph1* insertion site, on genomic DNA from *phdef-151* second-whorl organs and *star* and *wico* sepals and petals. The large fragment still contains the *dTph1* transposon inserted (expected size: 407 bp), while small fragments result from different events of *dTph1* excision (expected size: 115 bp) and were subsequently sequenced. **C)** The small *PhDEF* fragments from **B)** were sequenced in the second-whorl organs of flowers with a *phdef* (*n* = 2), *star* (*n* = 14), and *wico* (*n* = 14) phenotype. The nucleotidic sequence and predicted protein sequence are indicated, with stop codons represented by a *star*. Additional nucleotides or amino acids as compared to the wt sequences are indicated in red. *n*, number of independent reversion events where the same excision footprint was found; wt, wild-type.

To test this hypothesis, we characterized the *phdef-151* locus from in total 14 *star* and 14 *wico* independent reversion events (Fig. 2). For this, we amplified part of the *PhDEF* locus (Fig. 2A) and specifically sequenced the fragments resulting from *dTph1* excision in *phdef-151*, *star*, and *wico* second-whorl organs (Fig. 2, B and C). In *phdef-151*, the *dTph1*-excised alleles were always out of frame, with either 7 or 8 additional nucleotides as compared to the wt sequence. Due to a reading frame shift, both of these alleles are expected to produce an early truncated protein likely not functional (Fig. 2C), in line with the normal *phdef* mutant phenotype observed in these plants. In contrast, in both *star* and *wico* flowers, we could find either wt sequences (found 1 time and 3 times independently in *star* and *wico* flowers, respectively) or in-frame footprint alleles consisting of various additions of 6 nucleotides (alleles further named *PhDEF*+*6*, found 13 times and 11 times independently in *star* and *wico* flowers, respectively; Fig. 2C). These last insertions are predicted to result in proteins with 2 additional amino acids inserted toward the end of the DNA-binding MADS domain (Fig. 2C). Together, these results demonstrate that *wico* and *star* revertant flowers depend on the presence of an in-frame *def-151*-derived excision allele that partially restores petal development.

However, and in contrast to our initial expectations, there was no association between the sequence of the locus after excision and the phenotype of the flower, and both *star* and *wico* flowers could be found with a wt *PhDEF* excision allele or with an identical *PhDEF+6* allele (e.g. the 6-bp GTCTGG footprint allele was frequently found both in *wico* and *star* flowers). This indicates that the phenotypic difference between the *star* and *wico* flowers cannot be explained by a differently modified *PhDEF* sequence after *dTph1* excision. Secondly, since the *phdef* mutation is fully recessive (Vandenbussche et al. 2004), the presence of one transposon mutant allele combined with the wt revertant sequence normally should lead to wt flowers. Together, this implied that another molecular mechanism was causing the difference between *wico* and *star* flowers.

### The *wico* flowers are L1 periclinal chimeras

Excision of *dTph1* from a gene can occur at different times during plant development: if happening at the zygotic stage, then the whole plant will have a *dTph1*-excised allele. If excision occurs later, this will result in a genetic mosaic (chimera) with a subset of cells carrying the *dTph1* insertion at the homozygous state and others having a *dTph1*-excised allele. This typically leads to branches or flowers with a wt phenotype on a mutant mother plant (assuming a recessive mutation). Furthermore, since all plant organs are organized in clonally independent cell layers, excision can happen in one cell layer only, thereby creating a periclinal chimera, i.e. a branch or flower where cell layers have different genotypes (De Keukeleire et al. 2001; Frank and Chitwood 2016).

Analyzing the progeny of *wico* flowers suggested that they were periclinal chimeras, since the *wico* phenotype was not heritable (in consequence, they had to be maintained by cuttings of revertant branches). Instead, we found that the progeny of the *wico* flowers displayed a *phdef* mutant phenotype at a proportion close to 100%, undistinguishable from the parental *phdef-151* allele (Table 1). This suggested that the gametes generated by the *wico* flowers exclusively carried the mutant *phdef-151* allele, hence resulting in homozygous *phdef-151* mutants in the progeny. Gametes are exclusively derived from the L2 layer in flowering plants (Tilney-Bassett 1986), therefore indicating that L2-derived germ cells were homozygous mutant for *phdef-151* in *wico* flowers, which should result in a *phdef* phenotype if the epidermal tissue had the same genotype. This discrepancy suggested that the L1 layer of *wico* flowers was probably carrying a functional *PhDEF* allele.

**Table 1.** Progeny of the *star* and *wico* flowers after selfing

		Phenotype of the progeny (% of the total)		
		*phdef*	wt	Pink wt
**Parent flower**	*wico*-1	15 (94%)		1 (6%)^a^
	*wico*-2	14 (88%)	1 (6%)^a^	1 (6%)^a^
	*wico*-3	16 (100%)		
	*wico*-4	15 (94%)		1 (6%)^a^
	*wico*-5	16 (100%)		
	*wico*-6	12 (100%)		
	*wico*-7	12 (100%)		
	*star*-1	11 (46%)	4 (17%)	9 (38%)
	*star*-2	4 (25%)	4 (25%)	8 (50%)
	*star*-3	7 (29%)	5 (21%)	12 (50%)
	*star*-4	3 (19%)	3 (19%)	10 (63%)

Seven *wico* flowers and 4 *star* flowers have been selfed, and their progeny has been phenotyped and classified into *phdef*, wt, or pink wt phenotype. Summing the *star* progeny for the 4 parents gives 25 *phdef*, 16 wt, and 39 pink wt plants, which is not significantly different to a 1:1:2 ratio (chi-square test, *P* = 0.35).

^a^For *wico*, we found 4 plants with wt or pink wt flowers in the progeny, and all of them were linked to the presence of a de novo transposon excision from the *PhDEF* locus, restoring either a *PhDEF+6* (in the case of pink wt progeny) or a wt *PhDEF* (in the case of the wt progeny) allele.

**Figure 3.**
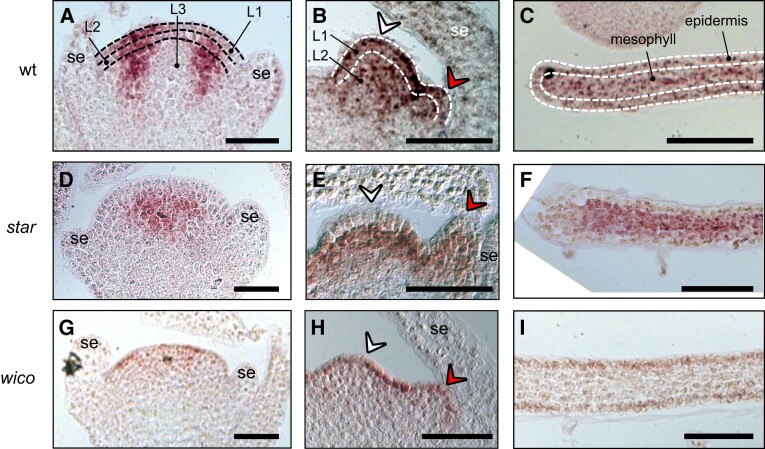
Localization of the *PhDEF* transcript in wt, *star*, and *wico* flowers by in situ hybridization. Longitudinal sections of wt **A to C)**, *star***D to F)**, and *wico***G to I)** flowers or young petals hybridized with a digoxigenin-labeled *PhDEF* antisense probe. At the earliest stage chosen **A, D, G)**, sepals are initiating and *PhDEF* is expressed in the future petal/stamen initiation domain. Note that if the section was not performed at the center of the flower, the *PhDEF* signal might artificially appear to be in the middle of the flower (as in **D**) whereas it is actually on its flanks. At the middle stage chosen **B, E, H)**, stamens (white arrowhead) and petals (red arrowhead) are initiating, and *PhDEF* is expressed in both primordia. The meristematic L1, L2, and L3 layers are indicated on the wt sections **A, B)**. *PhDEF* expression is also detected at the tip of young petal limb **C, F, I)**. The epidermis and mesophyll layers, derived from the previous L1 and L2 meristematic layers, are indicated on the wt section **C)**. se, sepals. Scale bar: 50 *µ*m.

To test this hypothesis, we localized the *PhDEF* transcript in *wico* flowers by in situ hybridization (Fig. 3; Supplemental Fig. S3). In wt flowers, the *PhDEF* transcript was first detected in the stamen initiation domain, then shortly after in incipient stamen and petal primordia (Fig. 3, A and B). At all stages observed, *PhDEF* expression appeared quite homogeneous in all cell layers of the organs, with a stronger expression in the distal part of the petal at later stages of development (Fig. 3C; Supplemental Fig. S3). In contrast, in *wico* flowers, *PhDEF* expression was restricted to the L1 and epidermis, all throughout petal development (Fig. 3, G to I; Supplemental Fig. S3). Therefore, we conclude that *wico* flowers are the result of an early *dTph1* excision event in a cell from the L1 meristematic layer, resulting in a chimeric flower expressing *PhDEF* only in the epidermis (L1-derived cells) of petals. *Wico* flowers are therefore L1 periclinal chimeras.

### The *star* flowers are L2 periclinal chimeras

Similarly, we analyzed the progeny of the *star* flowers, and the *star* phenotype was also not heritable and hence maintained by cuttings of revertant branches. The progeny of the *star* flowers with a *PhDEF+6* allele yielded 3 different phenotypic classes (in a proportion close to 1:1:2; Table 1): plants displaying a *phdef* phenotype, plants having wt flowers, and plants carrying flowers with a wt architecture but with altered pigmentation, further referred to as “pink wt” (Supplemental Fig. S4).

We genotyped the *PhDEF* locus in plants descendant from a single *star* parent and carrying flowers with a wt architecture (Supplemental Table S1). We found that all plants with a pink wt phenotype were heterozygous with an out-of-frame *phdef* allele and an in-frame *PhDEF+6* allele, while fully red wt flowers had in-frame *PhDEF+6* alleles at the homozygous state. This indicates that the PhDEF protein with 2 additional amino acids is not 100% fully functional, as it leads to a reduction in limb pigmentation when combined with an out-of-frame allele. The fact that it can ensure normal petal development when at the homozygous state indicates that this is dosage dependent. In summary, the segregation ratio shows that the *star* gametes carried either the *phdef-151* allele or an in-frame *PhDEF* allele at a 1:1 ratio, and hence, that the germ cells generating these gametes were heterozygous for these 2 alleles. This suggested that in *star* flowers, the L2 layer was carrying a functional *PhDEF* allele (either wt *PhDEF* or *PhDEF+6*) while the L1 layer was homozygous mutant for *phdef-151*.

In support of this, in *star* flowers, *PhDEF* expression was absent from the L1 and epidermis (Fig. 3, D to F; Supplemental Fig. S3). At the petal margins, underlying layers were also devoid of *PhDEF* expression (Fig. 3F), which likely corresponds to the restricted petal area where cells of L1 origin divide periclinally and invade the mesophyll (Satina and Blakeslee 1941). Therefore, we conclude that *star* flowers are the result of an early *dTph1* excision event in a cell from the L2 meristematic layer, resulting in a chimeric flower expressing *PhDEF* only in the mesophyll (L2-derived cells) of petals. *Star* flowers are therefore L2 periclinal chimeras. Considering the *star* and *wico* phenotypes, we can conclude that the petal epidermis is the main driver for limb morphogenesis (growth, shape, and pigmentation), while the mesophyll mainly drives tube morphogenesis (growth and shape).

### Non-cell-autonomous effects of layer-specific *PhDEF* expression on cell identity

**Figure 4.**
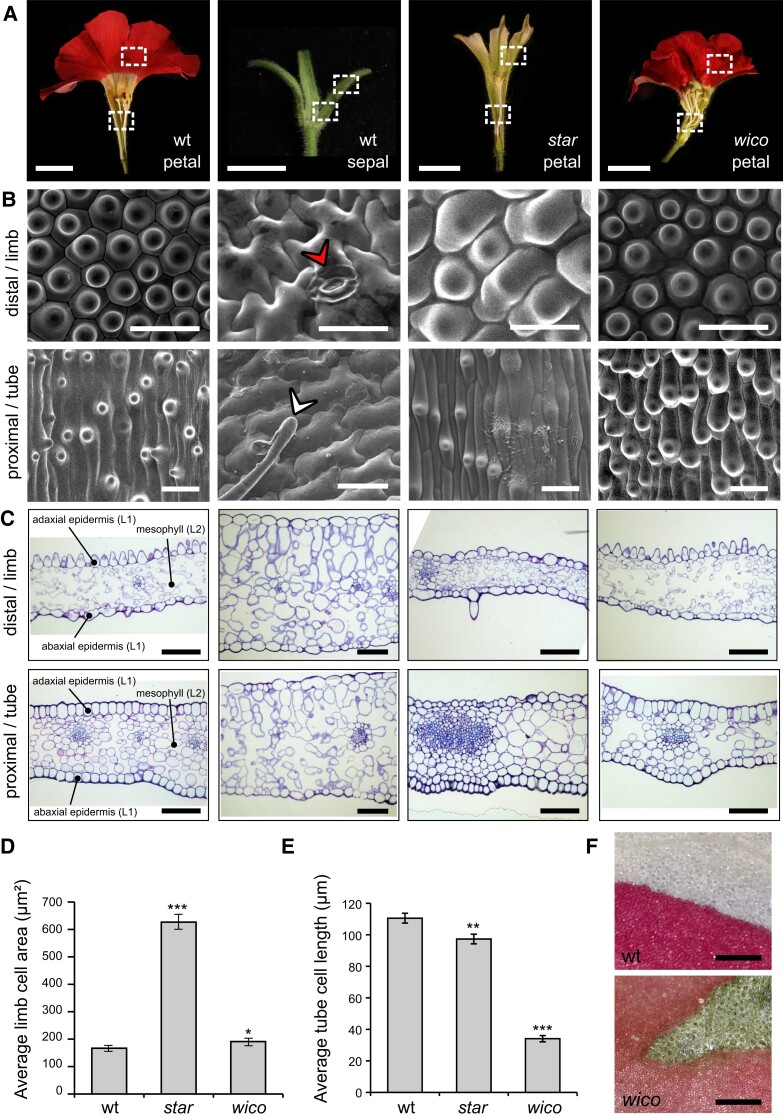
Epidermal and mesophyll cell identities in wt petals and sepals, and *star* and *wico* petals. **A)** From left to right: wt petals, wt sepals, *star* petals, and *wico* petals cut open longitudinally to show areas used for SEM and cross-sections. Petals were subdivided into limb and tube area, and sepals were subdivided into a distal and a proximal part, as shown by the dotted white rectangles. Scale bar: 1 cm. **B)** Representative scanning electron micrographs from the adaxial side of a wt petal, wt sepal, *star* petal, and *wico* petal (from left to right). The red arrowhead points to a stomata, and the white arrowhead points to a trichome. Scale bar: 30 *µ*m. **C)** Representative cross-sections from wt petals, wt sepals, *star* petals, and *wico* petals (from left to right) stained with toluidine blue. The adaxial and abaxial epidermis and the mesophyll are indicated on the wt petal sections. Scale bar: 100 *µ*m. **D)** Average limb cell area from the adaxial side of wt, *star*, and *wico* petals (*n* = 30 cells). Student’s *t* test with Welch correction, 2-sided (*P < 0.05, ***P < 0.005). Error bars represent ±SEM. **E)** Average tube cell length from the adaxial side of wt, *star*, and *wico* petals (*n* = 40 cells for wt and 45 cells for *star* and *wico*). Wilcoxon rank sum test, 2-sided (**P < 0.01, ***P < 0.005). Error bars represent ±SEM. **F)** Limb area from wt (top) and *wico* (bottom) petals, after their adaxial epidermis was manually peeled. For wt, the upper half of the picture shows the white underlying mesophyll. For *wico*, the green triangular area shows the green (chloroplastic) underlying mesophyll. Scale bar: 300 *µ*m.

Having determined the genetic basis of the *star* and *wico* phenotypes, we next wondered how layer-specific *PhDEF* expression affects the determination of cell identity, in the layer where *PhDEF* is expressed (cell-autonomous effect) but also in the layer devoid of *PhDEF* expression (non-cell-autonomous effect). For this, we observed petal adaxial epidermal cells by scanning electron microscopy (SEM) and mesophyll cells on petal cross-sections, in wt petals and sepals, and in *star* and *wico* petals (Fig. 4).

On the adaxial side of the wt petal (Fig. 4A), cells from the limb are round and conical as in many angiosperm petal limbs, while cells from the tube are elongated with a central cone (Fig. 4B; Cavallini-Speisser et al. 2021). In contrast, the adaxial epidermis of wt sepals (indistinguishable from *phdef-151* second-whorl organs) displays typical leaf-like features (Morel et al. 2019), with puzzle-shaped cells interspersed with stomata and trichomes (Fig. 4B). Epidermal cell identity can thus be clearly determined on the basis of cell shape. In *wico* petals, epidermal limb cells are conical, similar to wt cells from the same area, although marginally bigger (Fig. 4, B and D). In contrast, cells from the tube, albeit displaying a similar shape to wt cells, are strongly reduced in length (Fig. 4, B and E), suggesting that a defect in cell elongation is at least partly responsible for tube length reduction in *wico* petals.

In *star* petal tubes, epidermal cells have a similar appearance as in a wt petal tube but are slightly less elongated (Fig. 4, B and E). In contrast, epidermal cells from the *star* limb are slightly bulging cells, more or less roundish and about 3 times larger than wt conical cells (Fig. 4D). Pigmented revertant sectors on star flowers (resulting from an additional *dTph1* excision in the epidermis) allow the immediate comparison between *star* and wt epidermal cells on a single sample, confirming the difference in conical cell size, shape, and color (Supplemental Fig. S5). Moreover, the *star* limb adaxial epidermis occasionally forms trichomes (Supplemental Fig. S5), a feature that is normally not observed in the wt limb adaxial epidermis. Altogether, these observations suggest that epidermal cells from *star* limb have an intermediate identity between petal and sepal cells.

Mesophyll cell identity was investigated by analyzing petal cross-sections stained with toluidine blue (Fig. 4C). In the wt petal, mesophyll cells are loosely arranged, big and round in the tube, and small and elongated in the limb. Sepal mesophyll cells are bigger than petal mesophyll cells, and they display the typical leaf mesophyll organization with an upper palisade layer (elongated and parallel cells) and a lower spongy layer (dispersed cells). Hence, mesophyll cell size, shape, and tissue-level organization are characteristic features allowing to distinguish between sepal and petal mesophyll tissue.

In *star* petals, the mesophyll strongly resembles a wt petal mesophyll in its organization; however, cells are bigger and more densely packed in the tube, suggesting that *PhDEF* activity in the L2 layer is not entirely sufficient to specify normal mesophyll formation in the tube, which might be linked to the slightly reduced size of the tube of *star* flowers (Fig. 1L). In *wico* petals, mesophyll cells appeared very similar to wt, and their organization was clearly distinct from the one found in sepals since no palisade layer was observed. However, peeling the epidermis from *wico* limb revealed that the underlying mesophyll harbored chloroplasts, similar to a sepal mesophyll and in striking contrast with the white mesophyll of wt petal limb (Fig. 4F). Thus, the *phdef* mutant mesophyll in *wico* flowers has an intermediate identity between sepal and petal. In summary, our results show that for most features, *PhDEF* directs petal cell identity autonomously, and that nonautonomous effects also influence cell identity across layers. The interpretation of these effects is summarized in Supplemental Fig. S6. In contrast, the observation of *star* revertant sectors (Supplemental Fig. S5) revealed that cell identity is entirely defined autonomously within the epidermal layer, since a sharp transition in cell pigmentation, size, and shape is observed in these sectors (Supplemental Fig. S5). This suggests that different processes are at stake for cell–cell communication of petal identity across and within layers.

The physical nature of the non-autonomous effects that we identified remains unknown. Our in situ hybridization assays show that the mRNA of *PhDEF* is not mobile, but our attempts to localize the PhDEF protein by immunohistochemistry have been unsuccessful; hence, we do not know if the PhDEF protein itself might move between petal layers. Alternatively, and nonexclusively, other molecular players or mechanical signals might mediate information between layers.

### Transcriptome sequencing of *star* and *wico* petals

**Figure 5.**
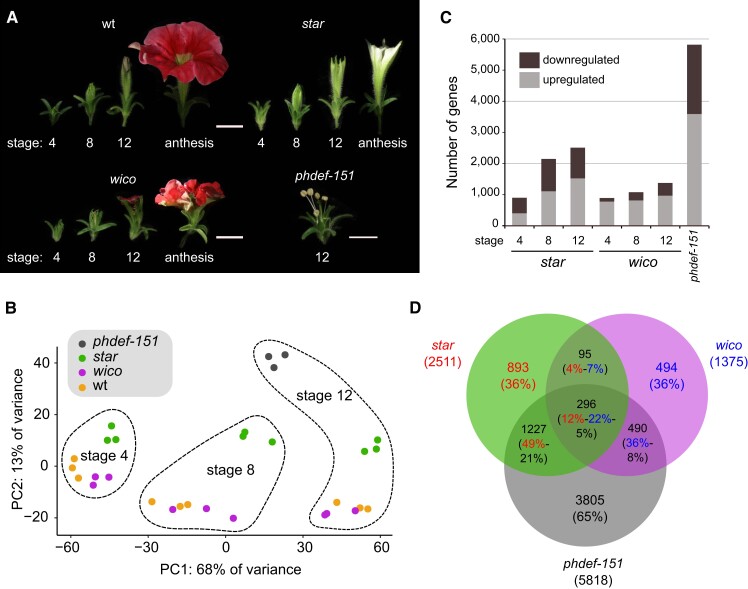
Gene differential expression in *star* and *wico* petals. **A)** Flowers from wt, *star*, *wico*, and *phdef-151* at stages 4, 8, and 12 (only stage 12 for *phdef-151*), whose petals or sepals were harvested for transcriptome sequencing. Flowers at anthesis are shown for comparison. Scale bar: 1 cm. **B)** Principal component analysis plot of the samples after analysis of variance with DESeq2, showing that the first principal component corresponds to the developmental stage and the second principal component corresponds to the genotype. **C)** Number of upregulated and downregulated genes in *star*, *wico*, and *phdef-151*, as compared to wt at the corresponding stages. **D)** Venn diagram recapitulating the number of DEGs in *star*, *wico*, and *phdef-151* petal samples at stage 12, as compared to wt, and their different intersections. Each sector contains the number of DEGs, and between parenthesis is the percentage of genes that it represents from the total number of DEGs in the corresponding sample, with a color code (red = percentage of DEGs from *star* samples/blue = from *wico* samples/black = from *phdef-151* samples).

To better understand the molecular basis for the *star* and *wico* phenotypes, we performed RNA-Seq on total petal tissue at 3 developmental stages, including wt and *phdef-151* samples (Fig. 5). We chose an early stage (stage 4 as defined in Reale et al. 2002), an intermediate stage (stage 8) when tube length is at half its final size, and a late stage (stage 12) before limb is fully expanded (Fig. 5A). For *phdef-151*, we only sequenced second-whorl sepal tissue at stage 12 (before anthesis). Principal component analysis showed that developmental stage is the first contributor to variation in gene expression, while genotype corresponds to the second axis of variation (Fig. 5B). All genotypes clustered separately except *wico* and wt samples which were highly similar at the 2 later stages. We analyzed one-to-one differential gene expression between mutant and wt samples with DESeq2 (Love et al. 2014), and we found on average 5,818 differentially expressed genes (DEGs) in *phdef-151*, as compared to 1,854 and 1,115 DEGs in *star* and *wico*, respectively, when averaging for all stages (Fig. 5C; Supplemental Data Set 1).

There were generally more upregulated genes than downregulated ones in mutant or chimeric genotypes, and the number of DEGs increased as development progressed in the petal in both *star* and *wico* (Fig. 5C). At stage 12, a large proportion of DEGs (58% to 61%) in *wico* or *star* petals were also differentially expressed in *phdef-151* (Fig. 5D), as expected since *wico* and *star* flowers are mutant for *PhDEF* in one cell layer. Genes uniquely differentially expressed in *star* or *wico* flowers represented 36% of DEGs for each, and only 16% to 29% of DEGs were jointly differentially expressed in *star* and *wico* flowers, consistent with the very different phenotypes of these flowers. These proportions indicate that the *star* and *wico* phenotypes are mostly subtended by the differential expression of sets of genes also differentially expressed in *phdef-151*, together with the differential expression of a unique set of genes for each genotype.

In *star* and *wico* petals, we found that *PhDEF* was downregulated about 2-fold at all stages (Supplemental Fig. S7), as expected since *PhDEF* is expressed in one cell layer only. In contrast, *PhTM6* was not differentially expressed in *star* and *wico* nor in *phdef-151* (Supplemental Fig. S7), as expected since this atypical B-class gene is mostly expressed in stamens and carpels and its upregulation depends on the C-function genes (Rijpkema et al. 2006; Heijmans et al. 2012). Unexpectedly, we observed that the B-class genes *PhGLO1* and *PhGLO2* were not downregulated in *wico* petals, and only modestly in *star* petals, although their expression was almost null in the *phdef-151* mutant (Supplemental Fig. S7). The fact that *PhGLO1* and *PhGLO2* expression does not strictly mirror the expression of *PhDEF* in *star* and *wico* petals, which is what we would have expected since the B-class heterodimers are known to activate their own expression, suggests that *PhGLO1* and *PhGLO2* expression is not entirely dependent on the B-class heterodimeric complexes, in particular in the epidermal layer of the petal.

### PhDEF directly binds in vivo to the terminator region of *AN2*, encoding a major regulator of petal pigmentation

The *star* and *wico* periclinal chimeras have revealed layer-specific roles of PhDEF in the establishment of petal identity and petal development. More specifically, the major layer-specific phenotypes that we have identified are petal pigmentation, conical cell formation and limb growth (controlled by the epidermal-specific expression of *PhDEF*), and tube growth (controlled by the mesophyll-specific expression of *PhDEF*). Therefore, our chimeras show the potential to further explore the exact nature of the link between layer-specific *PhDEF* activity and layer-specific phenotypes. As a proof of concept, we explored if PhDEF could directly control petal pigmentation in the limb epidermis. Pigmentation appeared to us as a trait of choice, since its regulatory and biosynthetic factors are well described, while this was not the case for the other traits mentioned above. Moreover, the absence of pigmentation in *star* petals, the restoration of pigmentation in L1-revertant sectors, and the phenotype of the pink wt flowers all converged to a direct link between *PhDEF* expression in the epidermis and petal pigmentation.

**Figure 6.**
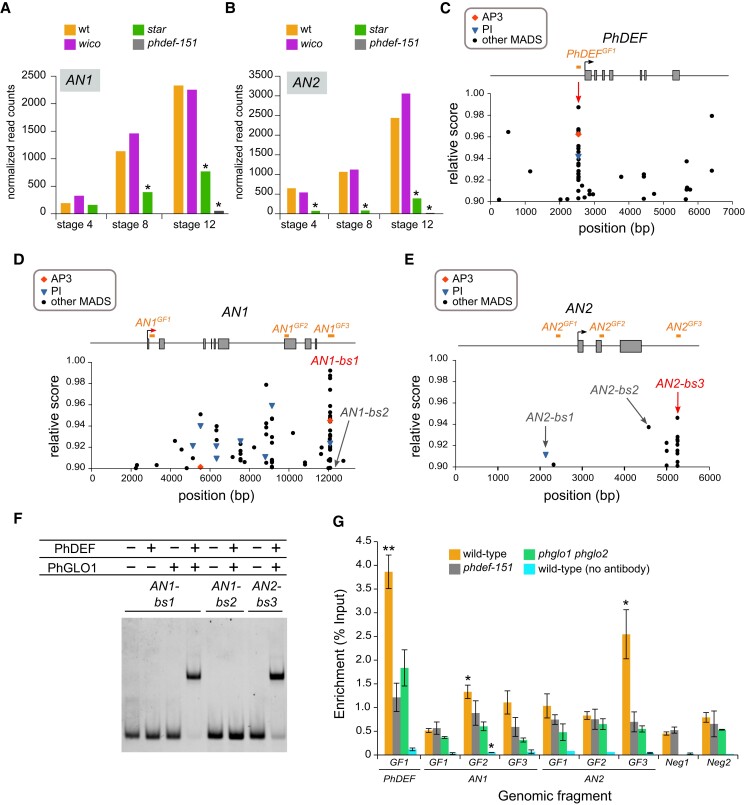
PhDEF binds to *AN2* regulatory region in vitro and in vivo. **A, B)** Expression (as normalized read counts calculated by DESeq2) of *AN1***A)** and *AN2***B)** in wt, *star*, *wico*, and *phdef-151* second-whorl organs at stage 4, 8, or 12. Stars indicate significant downregulation (log2FC < −1 and adjusted *P* < 0.01). **C to E)** Relative score profiles for AP3 (red diamond), PI (blue triangle), and all other MADS-box TFs (black dots) available on JASPAR, on the genomic sequences of *PhDEF***C)**, *AN1***D)**, and *AN2***E)**. The relative score is computed using the position weight matrix of each TF and is between 0 and 1; only relative scores higher than 0.9 are shown here. The gene model is represented above the score profile with exons as gray rectangles, the transcription start site as an arrow, and the gene model is aligned with the position of the predicted binding sites (bs). For *PhDEF*, the position of a putative CArG box, as explained in the main text, is indicated by a red arrow. The positions of the sites tested by gel shift in **F)** and Supplemental Fig. S8 are indicated: putative PhDEF binding sites (*AN1-bs1*, *AN2-bs1*, *AN2-bs2*, and *AN2-bs3*) and a negative control with a low predicted binding score (*AN1-bs2*). Sites indicated in red were bound in the gel shift assay, while sites indicated in gray were not bound. In orange, the GF tested by ChIP are depicted in **G)**. **F)** Representative electrophoretic mobility shift assay (EMSA) gel performed with a combination of in vitro translated PhDEF and/or PhGLO1 proteins, and Cy5-labeled *AN1-bs1*, *AN1-bs2*, or *AN2-bs3* DNA fragments, whose position is depicted in **C to E)**. Similar results were obtained in 5 additional independent assays for *AN1-bs1*, 2 additional independent assays for *AN2-bs3*, and 4 additional independent assays for *AN1-bs2*. **G)** Enrichment (as percentage of input) of binding of PhDEF to different genomic regions of the chromatin purified from wt, *phdef-151*, or *phglo1 phglo2* second-whorl organs at stage 8, after immunoprecipitation with an anti-PhDEF directed antibody. The control without antibody was performed on chromatin isolated from wt petals. The position of the GF tested is depicted in **C to E)**. Neg1 and Neg2 represent 2 negative control fragments located in the promoter region of genes not differentially expressed in the *phdef-151* mutant and present on different chromosomes than *PhDEF*, *AN1*, and *AN2*. For unknown reasons, the Neg1 control region could never be amplified in the *phglo1 phglo2* samples. *Star*s indicate a significant enrichment of test regions over the average of the 2 negative control regions for each chromatin sample (one-sided *t* test with Welch correction, *P < 0.05, **P < 0.005; *n* = 3 biological replicates for wt and *phdef-151* and 2 biological replicates for *phglo1 phglo2* and the control without antibody). Error bars represent ±SEM.

For this, we examined the 451 genes downregulated in both *phdef-151* and *star* samples (at any stage) but not differentially expressed in *wico* samples (Supplemental Data Set 2), and we found 23 anthocyanin-related genes in this gene set (Supplemental Fig. S7), out of a total of 42 in the whole genome, which constitutes an exceptionally high enrichment for this gene function (*P* < 0.001, Fisher's exact test). We paid particular attention to the genes possibly involved in the first steps of anthocyanin production, i.e. encoding proteins involved in the MBW complexes activating anthocyanin biosynthesis (AN1, AN2, AN4, AN11, JAF13, DPL, and PURPLE HAZE). We found that *AN1*, *AN2*, *DPL*, and *JAF13* were downregulated both in *phdef-151* and *star* samples (Supplemental Fig. S7 and Data Set 2). DPL is involved in the limb venation pattern (Albert et al. 2011; Zhang et al. 2021), and JAF13 has only a moderate contribution to limb pigmentation (Bombarely et al. 2016); therefore, we decided to focus our attention on the 2 major activators of anthocyanin biosynthesis, AN1 and AN2 (Fig. 6).

Indeed, the *an1* mutant has fully white petals, and the *an2* mutant has strongly reduced limb pigmentation (Quattrocchio et al. 1999; Spelt et al. 2000). Furthermore, AN2 was shown to act as an upstream activator of *AN1* since overexpressing *AN2* in petunia leaves is sufficient to activate *AN1* expression and for anthocyanins to accumulate (Quattrocchio et al. 1998; Spelt et al. 2000). We observed that both genes were already expressed at stage 4 of wt petal development, before any pigmentation is visible, and their expression levels strongly increased from stage 4 to stage 12, while both being strongly downregulated in *star* petals and *phdef-151* second-whorl organs but not in *wico* flowers (Fig. 6, A and B). *AN2* was expressed at higher levels than *AN1* at all stages, consistent with its most upstream role in the anthocyanin pigmentation pathway.

We aimed to test if PhDEF could directly bind to *AN1* and *AN2* genomic sequence, potentially to regulate their expression. For this, we first attempted to predict PhDEF binding on the genomic sequences of *AN1* and *AN2*. We used the high-quality TF binding profile database JASPAR (Sandelin et al. 2004; Fornes et al. 2020), using position weight matrices for each TF to compute relative binding scores that reflect in vitro binding preferences (Stormo 2013). The exact DNA-binding specificity of PhDEF has not been characterized, but that of its Arabidopsis homologs AP3 and PI has been (Riechmann et al. 1996b). However, since PhDEF DNA-binding specificity might be slightly different to those of AP3 and PI, we decided to predict binding for all MADS-box TFs available in JASPAR 2020, accounting for 23 binding profiles including those of AP3 and PI (Fornes et al. 2020). We hypothesized that sequences predicted to be bound by several MADS-box TFs were putative CArG boxes (the binding site for MADS-box proteins, whose canonical sequence is CC(A/T)_6_GG, but real binding sites show some variation to this consensus; Aerts et al. 2018).

As a validation of this strategy, we analyzed the genomic sequence of *PhDEF* and found a putative CArG box in the *PhDEF* promoter (visible by the presence of good predicted binding sites for several MADS-box proteins and therefore appearing as a clear black line in Fig. 6C). This CArG box has been validated in the literature: it is highly conserved between distantly related flowering plants (Rijpkema et al. 2006) and it was shown to be important for *AP3* petal-specific expression and for its autoactivation in Arabidopsis (Hill et al. 1998; Wuest et al. 2012) and for DEF function and binding to its own promoter in *Antirrhinum* (Schwarz-Sommer et al. 1992). We next applied this predictive approach to the genomic sequences of *AN1* and *AN2*. For *AN1*, we predicted a putative CArG box (*AN1-bs1*) with a very high score for several MADS-box proteins and for AP3 and PI in particular, in the terminator region (Fig. 6D). For *AN2*, we also predicted a putative CArG box (*AN2-bs3*), again in the terminator region of the gene (Fig. 6E), although its binding score was more modest in comparison to *AN1-bs1*. The sequence of *AN1-bs1* corresponds to a close-to-canonical CArG box (CTATATTTGG), and the sequence of *AN2-bs3* corresponds to a perfectly symmetrical canonical CArG box (CCATAATAGG).

To determine if PhDEF could indeed bind to *AN1-bs1* and *AN2-bs3* and potentially regulate *AN1* and *AN2* expression, we performed gel shift assays using in vitro translated PhDEF and/or PhGLO1 proteins (Fig. 6F). We found that, when incubating a 60-bp fragment containing *AN1-bs*1 in its center with either PhDEF or PhGLO1, no shift in migration was visible, indicating that neither protein could bind to this site alone. However, when incubating *AN1-bs1* with both PhDEF and PhGLO1 proteins, we observed a clear shift in migration, consistent with the obligate heterodimerization of these proteins necessary for DNA binding (Riechmann et al. 1996a). Similarly, a 60-bp fragment containing *AN2-bs3* in its center, incubated with PhDEF and PhGLO1 proteins, resulted in a clear shift in migration. In contrast, a control 60-bp fragment named *AN1-bs2*, located in the *AN1* terminator region but predicted to have a very low binding score (relative score under 0.8 both for AP3 and PI), was not bound by the PhDEF + PhGLO1 protein complex, showing that our assay was specific. Therefore, PhDEF, when dimerized with PhGLO1, is able to bind to sites in putative regulatory regions in *AN1* and *AN2*, suggesting that it might directly regulate the expression of these 2 genes.

Next, we tested if PhDEF could bind in vivo to genomic regions containing *AN1-bs1* and *AN2-bs3* by ChIP. We produced recombinant PhDEF protein devoid of its highly conserved MADS domain, to avoid cross-reactivity with other MADS-box proteins, and generated a polyclonal antibody against this truncated PhDEF protein. We performed the ChIP assay on second-whorl organs (petal or sepal) from wt, *phdef-151*, or *phglo1 phglo2* plants at an intermediate stage of development (stage 8). In wt petal samples, we found a significant binding enrichment for some of the genomic fragments (GF) that we tested, and in particular *PhDEF^GF1^* (Fig. 6G), containing the validated CArG box previously described (Fig. 6C), which is expected since PhDEF activates its own expression.

We also observed a significant binding enrichment in *AN2^GF3^* (Fig. 6G), containing the previously identified *AN2-bs3* binding site (Fig. 6E). In contrast, no strong enrichment was detected in the *AN1* GF containing the *AN1-bs1* strong in vitro binding site for PhDEF (AN1^GF3^). Our ChIP assay was specific, since no enrichment was detected for the *phdef-151* mutant nor for the *phglo1 phglo2* mutant (Fig. 6G). The *phglo1 phglo2* samples constitute an indirect control for PhDEF binding, since the PhDEF protein partners PhGLO1/PhGLO2 are absent, thereby indirectly preventing PhDEF binding on DNA. The fact that we do not detect any binding enrichment in these plants shows that our ChIP assay is robust. Therefore, we conclude that PhDEF binds to the terminator region of *AN2* in planta, and that PhDEF is a putative direct regulator of *AN2* expression in the petal epidermis.

## Discussion

In this work, we identified periclinal chimeras expressing the B-class MADS-box gene *PhDEF* in different cell layers of the flower. This layer-specific expression resulted in the correct development of subdomains of the petal only, showing that epidermal *PhDEF* expression mainly drives limb morphogenesis while its expression in the mesophyll is more important for tube morphogenesis. This indicates that cell layer–specific actions of PhDEF are different and contribute in a complementary fashion to overall petal development.

### Contribution of cell layers to mature petunia petals

The SAM of all flowering plants is organized in 3 independent layers. Generally, it is assumed that L1-derived cells form the epidermis, L2-derived cells produce the mesophyll and subepidermal tissue, and L3-derived cells generate the ground tissues (inner mesophyll, vasculature, and pith of the stem). However, there is variation to this general pattern between organs; for instance, Arabidopsis sepals, stamens, and carpels derive from these 3 layers, while petals derive from the L1 and L2 layers only (Jenik and Irish 2000). Moreover, the contribution of cell layers can vary between the same organ in different species: for instance, petals from *Datura stramonium* (member of the Solanaceae family like petunia) are derived from all 3 layers, in contrast to petals from Arabidopsis (Satina and Blakeslee 1941). Finally, even in an organ from a single species, cell layer contribution is not always homogeneous in different parts of the organ: in *Datura* petals, the L3 only participates in the vasculature at the base of the organ but does not contribute to the distal part of the petal, and the L1 invades the mesophyll at the petal edges (Satina and Blakeslee 1941).

In fact, the contribution of cell layers to mature organ organization can only be strictly assessed by clonal analysis, where one follows cell lineage using trackable cell-autonomous markers. In petunia, no clonal analysis has been performed so far; hence, one can only assume which cell layers participate in petal development based on clonal analyses performed in closely related species. In *Datura*, periclinal chimeras induced by colchicine treatment and refined histological observations have provided a detailed clonal analysis for cell layers in floral organs (Satina and Blakeslee 1941). The first visible event of petal initiation is a periclinal cell division from the L2 layer, and further growth of the petal depends primarily on cell divisions from the L2, both anticlinal and periclinal. The L3 layer only contributes to the vascular tissue at the very base of the petal. L1-derived cells form the epidermis by anticlinal divisions, except at the petal edges where periclinal divisions are observed, leading to L1-derived cells invading the mesophyll. Hence, the *Datura* petal is formed by all 3 layers with a major contribution of the L1 and L2 layers and a relative enrichment in L1-derived cells (by thinning of the mesophyll) progressing from the base toward the tip of the petal. In this work, we hypothesized that the petunia petal is formed similarly. Accordingly, we only obtained 2 phenotypic classes of periclinal chimeras, *star* and *wico*, suggesting that L3-specific *PhDEF* expression probably only leads to a *phdef* mutant phenotype.

The contribution of L1- and L2-derived tissues is heterogeneous in the petunia petal. Indeed, cross-sections in the middle of the petal tube indicate that the mesophyll is thick, with several layers of cells (Fig. 4C). The mesophyll tissue is quite dense in this part of the tube, with lacunae between cells being relatively small. In contrast in the limb, mesophyll cells are very small and interspersed with large lacunae. There is a general thinning of the mesophyll as we progress from the base of the petal toward its edges, whereas the epidermis always appears as a single layer of tightly connected cells. Therefore, the general contribution of cell lineages (L1 or L2 derived) to the petunia petal explains to a large degree the *star* and *wico* phenotypes. Indeed, the limb is mostly derived from the L1 layer, and therefore, recovery of this lineage in the *wico* flowers is sufficient to restore limb development. Similarly, the tube is composed of a much higher proportion of mesophyll than epidermis cells, and recovery of the mesophyll lineage in the *star* flowers is sufficient to restore tube development.

### Different cell layers drive tube and limb morphogenesis

The *star* and *wico* phenotypes revealed that in petunia petals, the epidermis is the main driver for limb morphogenesis while the mesophyll is the main driver for tube morphogenesis. The epidermis has been proposed to be the layer in control of organ morphogenesis, since it is a layer under tension that restricts growth of the underlying inner tissues that tend to expand (Kutschera and Niklas 2007). In particular, epidermal expression of the brassinosteroid receptor BRI1 (BRASSINOSTEROID INSENSITIVE 1) is sufficient to restore normal leaf morphogenesis in a *bri1* mutant (Savaldi-Goldstein et al. 2007). Similarly, the expression of the auxin transporter PIN1 (PIN-FORMED 1) in the L1 of the SAM is sufficient to restore normal phyllotaxis in a *pin1* mutant (Kierzkowski et al. 2013). However, pieces of evidence suggest that organ inner layers can have an active role in morphogenesis: for instance, mesophyll-specific expression of *ANGUSTIFOLIA* (*AN*) is sufficient to restore normal leaf width in the Arabidopsis *an* mutant (Bai et al. 2010); leaf shape is controlled by the L2- and L3-derived tissues in *Nicotiana glauca* (McHale and Marcotrigiano 1998); and the leaf mesophyll is the main player for leaf flatness in Arabidopsis (Zhao et al. 2020). Moreover, expressing *BRI1* in the root phloem also restores *bri1* plant dwarfism (Graeff et al. 2020). The contribution of cell layers to organ morphogenesis is thus a complex process that varies between organs, species, and the genetic systems investigated.

Our work has confirmed that the petunia petal has a modular structure, since tube and limb can develop relatively independently from each other in the *star* and *wico* flowers. This modularity is consistent with previous observations in the literature (described in the Introduction) and in line with the different ecological roles of the tube and the limb for the interaction with pollinators. Our results highlight that a homeotic factor, PhDEF, can participate in the establishment of this modular structure. Indeed, although PhDEF is normally present in all cell layers of the wt petal, its action in the different cell layers is mainly responsible for tube or limb development. This provides a possible mechanism, at the tissue level, for the establishment of the modular structure of petunia petals by homeotic genes. It also contributes to the understanding of how homeotic genes can specify at the same time the overall identity of an organ and the coordinated development of its different functional modules.

One may wonder if our findings apply outside of petunia flowers. In snapdragon and Arabidopsis flowers, periclinal chimeras for orthologs of *PhDEF* (*DEF* and *AP3*, respectively) or *PhGLO1/PhGLO2* (*GLO* and *PI*, respectively) have been previously obtained (Bouhidel and Irish 1996; Perbal et al. 1996; Efremova et al. 2001; Jenik and Irish 2001; Vincent et al. 2003; Urbanus et al. 2010b). In snapdragon, expression of *DEF* only in the L1 layer largely restores petal development, particularly in the limb, in contrast to the L2/L3-specific *DEF* or *GLO* expression which causes reduced limb growth (Perbal et al. 1996; Efremova et al. 2001; Vincent et al. 2003). Petals are fused into a tube in snapdragon flowers, but the tube is much more reduced than in petunia; hence, conclusions on tube length restoration in the chimeras were not drawn by the authors. However, in light of our results, it is clear that snapdragon chimeras expressing *DEF* or *GLO* in the L2/L3 layers restore tube development to a higher degree than limb development, similar to what we observed. In Arabidopsis that has simple and unfused petals, petal size was never fully restored when *AP3* was expressed in one cell layer only, while petal shape was normal (Jenik and Irish 2001; Urbanus et al. 2010b); in contrast, epidermal expression of *PI* was sufficient to restore normal petal development (Bouhidel and Irish 1996). Therefore, it seems that the contribution of different cell layers to petal development varies across species and depending on the petal identity gene under investigation.

### Autonomous and non-autonomous effects of *PhDEF* expression on petal traits

Our study revealed that petal traits are affected differently by layer-specific *PhDEF* expression (Supplemental Fig. S6). For instance, epidermal pigmentation is a clearly autonomous trait, since *star* petals are not pigmented except when wt revertant sectors arise. On the contrary, epidermal cell shape appears to behave as a partially autonomous trait since *star* epidermal cells have an intermediate phenotype between wt petal conical cells and sepal epidermal cells. Finally, organ size and shape are specified non-autonomously in subdomains of the petal: *PhDEF* expression in the L1 or L2 is sufficient to specify correct shape of the limb or correct size and shape of the tube, respectively, suggesting that in these petal domains, layer-specific *PhDEF* expression is sufficient to signal cells from the other layer to grow normally.

The mechanisms for this interlayer communication remain unknown. Our in situ hybridization assays show that the *PhDEF* mRNA is not mobile between layers, but our attempts to detect the PhDEF protein in petal tissue by immunohistochemistry have been unsuccessful; therefore, we do not know if the PhDEF protein itself might be moving between layers, which would be the simplest mechanistic explanation for the non-autonomous traits that we observe. Indeed, in *Antirrhinum* petals expressing *DEF* in the L2/L3 layers, the DEF protein was found in small amounts in the epidermis, and it is likely why petals from these chimeras are faintly pigmented (Perbal et al. 1996; Vincent et al. 2003). This indirectly suggests that no such movement occurs in the *star* petals that are mostly white. In contrast, Arabidopsis AP3 and PI GFP fusion proteins are unable to move between cell layers, although they can move within the epidermal layer (Urbanus et al. 2010a, 2010b). In any case, even if the PhDEF protein would move between layers in our chimeric flowers, it is likely to be in small amounts only and possibly at restricted stages of development; otherwise, both flower types would have a wt phenotype. Therefore, it is unlikely to be the sole reason for tube and limb correct development in the *star* and *wico* flowers.

Alternatively, the non-autonomous effects that we observed might be triggered by mechanical signals transmitted between layers. For instance, in *star* flowers, normal growth of the mesophyll could merely drag along epidermal cells, since cells are connected by their cell walls, which could be sufficient to trigger their expansion and division. Other features, like conical cell shape, might be directly influenced by mechanical signals. Indeed, conical cells are shaped by a circumferential microtubule arrangement controlled by the microtubule-severing protein KATANIN, and altering this arrangement affects conical cell shape (Ren et al. 2017). Microtubule arrangement responds to mechanical signals (Hamant et al. 2008), which are likely to be transmitted between layers. Therefore, it is possible that the formation of bulging cells in the *star* epidermis is merely triggered by mechanical signals from the growing underlying layer, independent of any petal identity specifier, as was recently evidenced from the observation of conical-like bulges on the hypocotyl of the tubulin kinase mutant *nek6* (Takatani et al. 2020). The molecular or physical nature of the signals involved in communication between layers remains to be explored in full depth.

### Toward the gene regulatory networks of petal development

Our *star* and *wico* material granted the opportunity to explore the gene regulatory networks driving petal development in petunia, more specifically by decoupling on the one hand tube versus limb development and epidermis versus mesophyll development on the other. However, these effects are confounded in our data set, since both epidermis and limb development are affected in *star* flowers, whereas both mesophyll and tube development are affected in *wico* flowers. Further analyses, such as sequencing the transcriptome from *star* and *wico* limb and tube tissues separately, would help uncouple these effects, but it is not easy to clearly separate these different domains during early stages of development, which are crucial stages for petal morphogenesis. Spatial transcriptomics techniques, such as single-cell RNA-Seq, would be ideal to precisely dissect transcriptional changes between layers and domains of the petal at young developmental stages.

Still, we exploited our transcriptomic data set by focusing our analysis on anthocyanin-related genes, because the molecular link between the early establishment of petal identity by homeotic TFs, such as PhDEF, and the late establishment of petal maturation traits, such as anthocyanin accumulation, was unknown. For this, we examined the presence of anthocyanin-related genes among genes downregulated both in *star* and *phdef-151* samples but not differentially expressed in *wico* samples. We found a very strong enrichment of anthocyanin-related genes in this data set, suggesting that the initial triggering event for most of the anthocyanin biosynthesis pathway was missing in *star* flowers.

Finally, we investigated the direct link between PhDEF and petal pigmentation and found that, in vitro, the PhDEF + PhGLO1 protein complex directly binds to predicted binding sites in the regulatory regions of *AN1* and *AN2*. We confirmed that PhDEF binds to the corresponding genomic region of *AN2 in planta* by ChIP, but not for *AN1*, confirming that in vitro binding does not necessarily imply in vivo binding, the last being strongly influenced by the local chromatin landscape. The binding site of PhDEF that we identified on *AN2* (*AN2-bs3*) lies in the terminator region of the gene (and the next gene on the chromosome is more than 100 kb away), which was surprising since around 80% of MADS binding sites are located within the 3 kb promoter region of their target genes (Aerts et al. 2018). However, the presence of a binding site in the terminator region is still compatible with an activating role in transcription, through DNA looping to the promoter (Jash et al. 2012) or by promoting transcription termination and reinitiation (Wang et al. 2000). Other putative CArG boxes in the genomic region of *AN2* are *AN2-bs1*, located 866 bp upstream the ATG in the promoter region, and *AN2-bs2*, located 62 bp downstream the STOP codon in the 3′UTR region. Both have noncanonical CArG box sequences (GAAAAGTAG for *AN2-bs1* and TCTTTTTTAA for *AN2-bs2*) and were not bound in our gel shift assay (Supplemental Fig. S8). Still, it is possible that regulators other than MADS-box TFs could form protein complexes with PhDEF and mediate looping to the promoter region of *AN2*. The precise mechanism by which PhDEF might activate *AN2* transcription remains to be uncovered.

When aligning Petunia *AN2* sequences, we found that *AN2-bs3* lies in a globally nonconserved region of the gene (Supplemental Fig. S8), and *AN2-bs3* is only conserved in *Petunia inflata*, one of the likely original parents of *P. × hybrida* (Bombarely et al. 2016). However, *cis*-regulatory elements are very fluid, and their sequences can change rapidly in short evolutionary times, without the gene regulation being necessarily lost (see for instance Schmidt et al. 2010; Krieger et al. 2022). Moreover, petal pigmentation is an extremely labile trait, and even within the *Petunia* genus, it has been lost and regained at least 2 times independently (Quattrocchio et al. 1999; Esfeld et al. 2018; Berardi et al. 2021). Therefore, the fact that *AN2-bs3* is not largely conserved does not necessarily imply that it is an unimportant site for *AN2* regulation in *P. hybrida*.

The fact that we detected strong *in planta* binding of PhDEF to *AN2*, together with the fact that *AN2* expression is strongly downregulated in the *phdef-151* transcriptome, suggests that PhDEF is a good candidate to directly activate *AN2* expression in the petal. Ectopic expression of *AN2* in petunia leaves is sufficient to trigger anthocyanin accumulation in this tissue, by inducing *AN1* expression among others (Quattrocchio et al. 1998; Spelt et al. 2000). Therefore, if PhDEF indeed activates *AN2* expression, it should be sufficient to launch the whole pigmentation pathway in the wt petal limb. However, to fully support this conclusion, functional tests on the role of PhDEF binding to *AN2-bs3* in regulating *AN2* expression should be conducted. A direct link between petal identity and pigmentation has yet to be established, although genetic evidence in orchid flowers strongly implied that different B-class protein heteromeric complexes are responsible for specific pigmentation spots in the different petal types, but physical binding of these B-class protein complexes on pigmentation genes was not tested (Hsu et al. 2021). The direct target genes of B-class proteins have been identified by ChIP-Seq and transcriptomic analyses in Arabidopsis (Wuest et al. 2012), but this species has unpigmented petals, thereby preventing us to draw any possible link between petal identity and pigmentation. The petunia petal is the ideal system to test this direct link, and our results suggest that PhDEF might be the direct link between petal identity and its epidermal pigmentation.

## Materials and methods

### Plant materials, growth conditions, and plant phenotyping

The *phdef-151* plants were obtained from the *P. × hybrida* W138 line and were grown in a culture room in long-day conditions (16 h light 22 °C; 8 h dark 18 °C; 75-WValoya NS12 LED bars; light intensity: 130 *μ*mol/m^2^/s). Hundreds of *phdef-151* flowers were observed over several years, and all of them show the same phenotype, also identical to the *def-1* and *green petal* (*gp*) mutant flowers (de Vlaming et al. 1984; van der Krol et al. 1993). The *wico* and *star* flowers were repeatedly obtained from several different *phdef-151* individuals and were maintained by cuttings. For this, branches where several *star* or *wico* flowers were already visible were cut into a ca. 5-cm-long segment, large flowers and leaves were removed, and the branch segment was planted into an hydrated Jiffy peat soil pellet (Jiffy Products International AS, Norway). When roots became visible on the outside of the pellet, it was transferred into the soil. Plant and flower pictures were obtained with a CANON EOS 450D camera equipped with objectives SIGMA 18 to 50 mm or SIGMA 50 mm. To measure tube length, the flower was cut longitudinally and photographed from the side. To measure limb area, the limbs were flattened as much as possible on a glass slide covered with transparent tape and photographed from the top. The photographs were used to measure D1 and D2 lengths and limb area with ImageJ.

### Genotyping

Extraction of genomic DNA from young leaf tissue was performed according to Edwards et al. (1991). The region spanning the *dTph1* insertion site in *PhDEF* was amplified using primers MLY0935/MLY0936 (Supplemental Table S2). PCR products were separated on a 2% (w/v) agarose gel, and fragments of interest were purified using the NucleoSpin Gel and PCR Clean-up kit (Macherey-Nagel) and sequenced with Eurofins SupremeRun reactions.

### In situ RNA hybridization

Floral buds from wt, 2 *wico*, and 1 *star* lines were fixed overnight in FAA (3.7% [v/v] formaldehyde, 5% [v/v] acetic acid, and 50% [v/v] ethanol), cleared in Histo-clear, and embedded in paraffin to perform 8 *µ*m sections. *PhDEF* cDNA sequence was amplified from wt petunia inflorescence cDNAs with primers MLY1738/MLY1739 (Supplemental Table S2), generating a 507 bp fragment excluding the part encoding the highly conserved DNA-binding domain. The digoxigenin-labeled RNA probe was synthesized from the PCR fragment by in vitro transcription, using T7 RNA polymerase (Boehringer Mannheim). RNA transcripts were hydrolyzed partially for 42 min by incubation at 60 °C in 0.1 M Na_2_CO_3_/NaHCO_3_ buffer, pH 10.2. Later steps were performed as described by Cañas et al. (1994). For imaging, slides were mounted in Entellan (Sigma) and imaged with a Zeiss Axio Imager M2 light microscope equipped with a Zeiss Axio Cam HRc camera.

### Petal cross-sections

Small pieces (around 5 mm^2^) of tissue were harvested from the proximal and distal parts of wt mature sepals and from the tube and limbs of wt, *star*, and *wico* mature petals. Samples were fixed overnight in FAA (3.7% [v/v] formaldehyde, 5% [v/v] acetic acid, and 50% [v/v] ethanol) and dehydrated in an ethanol series. Preinfiltration was performed in a 1:1 mixture of ethanol:Technovit 7100 (Electron Microscopy Sciences) for 4 h under light agitation, then overnight in a 1:3 ethanol:Technovit 7100 mixture. Infiltration was performed in the infiltration solution for 1.5 h under vacuum, then for 1 night followed by 1 additional week. Samples were arranged in the molds with the polymerization solution for 2 h at room temperature, then mounted with the Technovit 3040 resin to relieve the blocks from the molds. Blocks were sectioned with a microtome to generate 3 to 7-*µ*m-thick sections. Slides were incubated for 10 min in a 0.1% (w/v) toluidine blue solution and imaged with a Zeiss Axio Imager M2 light microscope equipped with a Zeiss Axio Cam HRc camera.

### SEM

Scanning electron micrographs were obtained with a HIROX SH-1500 bench top environmental scanning electron microscope equipped with a cooling stage. Samples were collected and quickly imaged to limit dehydration, at −5 °C and 5 kV settings. For cell area and length measurements, pictures were taken from 3 petal tubes and 3 petal limbs from different wt, *star*, and *wico* flowers. For each sample, 3 pictures were taken and 5 cells (for the tube) or 10 cells (for the limb) were measured for each picture. Measures were performed with ImageJ by manually drawing the outline or length of the cells.

### RNA-Seq

Petal tissue was collected at 1 Pm from several plants stemming from a single *star* line, a single *wico* line, and several individual wt plants (progeny of a single *star* flower) and *phdef-151* plants (progeny of the same *star* flower). Tube length was macroscopically measured to compare stages, the corolla was cut open, and stamens were removed as much as possible from the corolla by pulling on the filaments fused to the tube. One biological replicate contains total petal tissue from 2 flowers. Tissue was ground in liquid nitrogen, and RNA was extracted with the Spectrum Plant Total RNA Kit (Sigma) including on-column DNase digestion (Sigma). RNA integrity and quantity were determined by a Bioanalyzer RNA 6000 Nano assay (Agilent). Libraries were prepared with poly-A enrichment, and single-end 75-bp sequencing was performed on a NextSeq 500 platform (Illumina). Sixteen to 23 million reads were recovered per library. Reads were checked for quality with FastQC v0.11.4 (https://www.bioinformatics.babraham.ac.uk/projects/fastqc/), and adaptors and low-quality ends were trimmed with Cutadapt v 1.16 (Martin 2011) and custom Perl scripts. The reference genome sequence used for transcriptome analysis is the *Petunia axillaris* v1.6.2 HiC genome published in Bombarely et al. (2016) and further scaffolded by HiC by DNAzoo (Dudchenko et al. 2017, 2018); gene annotations were transferred from the published assembly to the HiC-scaffolded version using Blat (Kent 2002), Exonerate (Slater and Birney 2005), and custom Perl scripts. In the rare cases when gene annotations from the published genome mapped to several regions in the HiC-scaffolded genome, these different putative genes were identified by a letter added at the end of the gene identifier (for instance Peaxi162Scf00179g00121a). The complete set of reads was mapped on the reference genome sequence using HISAT2 v2.2.1 (Kim et al. 2015) to identify splicing sites, before performing mapping sample per sample. Reads per gene were counted using FeatureCounts v1.5.1 (Liao et al. 2014). DESeq2 version 3.12 (Love et al. 2014) was used with R version 4.0.3 to perform the principal component analysis and the differential gene expression analysis. Genes having less than 10 reads in the sum of all samples were considered as nonexpressed and discarded. Genes were considered to be differentially expressed if log2FoldChange > 1 or <−1 and *P*-adjusted value < 0.01. The bioinformatic pipeline for annotation transfer, read cleaning, splicing site discovery, read mapping, and preliminary DESEq2 results can be found at gitbio.ens-lyon.fr/rdp/petunia_star_wico_rnaseq. Venn diagrams were built with InteractiVenn (Heberle et al. 2015). Due to the automatic gene name annotation pipeline used in Bombarely et al. (2016) based on homology with tomato (*Solanum lycopersicum*) proteins, many of the previously characterized petunia genes have not been annotated according to their first described name, making interpretation of some of the RNA-Seq results less straightforward. We have manually added annotations of 42 genes from the anthocyanin biosynthesis pathway based on the Supplementary Note 7 from Bombarely et al. (2016), and 31 type-II MIKC-C MADS-box genes based on previous studies from the literature; these annotations can be found in Supplemental Data Set 1 of this manuscript. We noticed that the gene annotations from 3 major pigmentation genes, *DFR* (*DIHYDROFLAVONOL-4-REDUCTASE*, Peaxi162Scf00366g00630), *CHSa* (*CHALCONE SYNTHASE a*, Peaxi162Scf00047g01225), and *PH1* (Peaxi162Scf00569g00024), were lost during the gene annotation transfer procedure, because they lie in regions of the genome that are still poorly resolved. Therefore, we manually searched the position of these transcripts in the HISAT2 output and we were able to map part of the *DFR* and *CHSa* genes to 2 small scaffolds, while *PH1* position was not found. We added the transcript positions of *DFR* and *CHSa* in the gtf/gff files before running FeatureCounts. The read counts for *DFR* and *CHSa* reported in Supplemental Fig. S7 are therefore an underestimation of their actual expression levels, since we miss part of the genes.

### Prediction of MADS-box TF binding sites

Genomic sequences from *AN1*, *AN2*, and *PhDEF* from the *P. × hybrid*a R27 line, starting 3 kb upstream the START codon and ending 1 kb downstream the STOP codon, were scanned with all MADS-box TF matrices included in the JASPAR 2020 database (http://jaspar.genereg.net), only removing matrices from AGL42 and AGL55 which are much shorter than the other matrices and therefore yield much higher scores. Relative scores above 0.86 were plotted against their genomic position.

### Electrophoretic mobility shift assays

CDS sequences from *PhDEF* and *PhGLO1* were amplified from *P. × hybrida* R27 inflorescence cDNAs with primers MLY2382/MLY2383 and MLY2384/2385, respectively (Supplemental Table S2), and cloned into the in vitro translation vector pSPUTK (Stratagene) by NcoI/XbaI restriction. From these vectors, the PhDEF and PhGLO1 proteins were produced with the TnT SP6 High-Yield Wheat Germ Protein Expression System (Promega) according to the manufacturer's instructions. The terminator regions from *AN1* (0.8 kb) and *AN2* (1 kb), and part of the promoter region of *AN2* (1.2 kb), were amplified from *P*. × *hybrida* R27 genomic DNA with primers from Supplemental Table S2 and cloned into pCR-BluntII-TOPO (Thermo Fisher). Binding sites were amplified from these plasmids with primers listed in Supplemental Table S2, with the forward primer labeled with Cy5 in 5′. The labeled DNA was purified and incubated with the TnT in vitro translation mixture as described in Silva et al. (2016) before loading on a native acrylamide gel.

### PhDEF protein and antibody production

The *PhDEF* truncated cDNA (without the sequence coding for the MADS domain) was chemically synthesized with optimization for expression in *Escherichia coli* and cloned into a pT7 expression vector by ProteoGenix (www.proteogenix.science). The expected PhDEF protein starts at amino acid 60 (PSITT…) and ends at the last amino acid of the sequence (…FALLE), and a 6xHis tag was added at the N-terminal part of the protein. The 6xHis-PhDEF protein was purified by affinity column with a nickel resin under denaturing conditions (8 M urea) by ProteoGenix. The purified protein was injected in 2 rabbits for immunization by ProteoGenix, to generate PhDEF-directed polyclonal antibodies that were purified by affinity against the antigen. Both lots of purified antibodies were validated by immunoblot in petal or sepal tissues from wt, *phdef-151*, and *phtm6* samples.

### ChIP

One biological replicate comprises the full corolla from 2 flowers (wt), second-whorl sepals from 3 flowers (*phdef-151*), or second-whorl sepals from 3 to 4 flowers (*phglo1 phglo2*), and the full experiment was performed for 3 biological replicates for wt and *phdef-151* and 2 biological replicates for *phglo1 phglo2*. Samples at stage 8 were collected and ground in liquid nitrogen. Ground tissue was resuspended into 10 mL fixation buffer (10 mM HEPES pH 7.6, 0.5 M sucrose, 5 mM KCl, 5 mM MgCl_2_, 5 mM EDTA pH 8, Complete Protease Inhibitor Cocktail [Merck], and 14 mM 2-mercaptoethanol), and a double cross-linking was performed at room temperature (1 h with disuccinimidyl glutarate at 2.5 mM with gentle shaking and 5 min with formaldehyde 1% [v/v]). Cross-linking was stopped by adding glycine at 200 mM, and samples were put directly on ice. Cells were lysed with a 40-mL Dounce tissue grinder (Duran Wheaton Kimble), Triton X-100 was added at 0.6% (w/v), and the lysate was filtered subsequently through 100- and 40-*µ*m nylon meshes to recover nuclei. Nuclei were pelleted for 10 min at 3,000 × *g* at 4 °C, and the pellet was resuspended in 300 *µ*L of cold nuclear isolation buffer (i.e. fixation buffer without 2-mercaptoethanol), carefully deposited on 600 *µ*L of a 15% Percoll solution (15% [v/v] Percoll, 10 mM HEPES pH 8, 0.5 M sucrose, 5 mM KCl, 5 mM MgCl_2_, and 5 mM EDTA pH 8) and centrifuged for 5 min at 2,000 × *g* at 4 °C. The pellet was resuspended into 900 *µ*L of cold nuclear lysis buffer (50 mM Tris-HCl pH 7.5, 0.1% [w/v] SDS, and 10 mM EDTA pH 8) to lyse the nuclei, and chromatin was sonicated twice for 15 min with a Covaris S220 sonicator (peak power 105, duty factor 5, and cycles/burst 200 for 900 s). For each sample, 25 *µ*L of magnetic protein-A Dynabeads and 25 *µ*L of magnetic protein-G Dynabeads (Invitrogen) were washed twice with 100 *µ*L of cold ChIP dilution buffer (15 mM Tris-HCl pH 7.5, 150 mM NaCl, 1% [w/v] Triton X-100, and 1 mM EDTA pH 8) using a magnetic rack (MagRack 6, Cytiva). Beads were mixed with 2.5 *µ*g of anti-PhDEF antibody and 1.8 mL of cold ChIP dilution buffer and incubated for 2 h at 4 °C on a rotating wheel. Sonicated chromatin was centrifuged for 5 min at 15,000 × *g* at 15 °C, and 25 *µ*L of supernatant (for wt samples) or 50 *µ*L of supernatant (for *phdef-151* and *phglo1 phglo2* samples) was added to the mix of beads and antibody and incubated overnight at 4 °C on a rotating wheel. Beads were washed twice (1 quick wash and 1 long wash with 15-min incubation on a rotating wheel) with each of the following buffers: low salt wash buffer (0.1% [w/v] SDS, 1% [w/v] Triton X-100, 2 mM EDTA pH 8, 20 mM Tris-HCl pH 8, and 150 mM NaCl), high salt wash buffer (0.1% [w/v] SDS, 1% [w/v] Triton X-100, 2 mM EDTA pH 8, 20 mM Tris-HCl pH 8, and 500 mM NaCl), LiCl wash buffer (0.25 M LiCl, 1% [v/v] NP40/Igepal, 1% [w/v] deoxycholate, 1 mM EDTA pH 8, and 20 mM Tris-HCl pH 8), and TE buffer. Elution was performed twice with 250 *µ*L of elution buffer (0.1 M NaHCO_3_ and 1% [w/v] SDS) at 65 °C. IP and input samples were decrosslinked overnight at 65 °C by adding NaCl at 200 mM, then incubating for 2 h at 42 °C with 20 *µ*g proteinase K in 10 mM EDTA pH 8 and 40 mM Tris-HCl pH 6.5. DNA was purified with phenol:chloroform:isoamyl alcohol (25:24:1) followed by chloroform:isoamyl alcohol (24:1) and precipitated with ethanol at −20 °C, and the pellet was washed with ethanol 70%. The dry pellet was recovered in 50 *µ*L TE, and 1 *µ*L was used for each qPCR reaction, which was performed in technical triplicates for each biological replicate (3 for wt and *phdef-151* and 2 for *phglo1 phglo2* and the control without antibody). The qPCR reaction was performed with 1X FastStart Universal SYBR Green (Merck) and 0.3 *µ*M primer mix (Supplemental Table S2), for 40 cycles (15 s at 95 °C and 1 min at 60 °C) in a QuantStudio 6 Flex instrument (Thermo Fisher). Percentage of input (enrichment) was calculated as 100* e^(CtIN − log2(DF) − CtIP)^, with *e* the efficiency of the primer pair, CtIN the average Ct value for the input sample, DF the dilution factor, and CtIP the average Ct value for the IP sample, as described in Solomon et al. (2021). The significance of the enrichment was evaluated with a 1-tailed *t* test comparing the enrichment of the test region to the average of the enrichments of the 2 negative regions.

### Sequence alignments

The genomic sequences (3 kb upstream of the transcription starting site and 1 kb downstream of the STOP codon) of *AN2* from Solanaceae species were retrieved by blasting the *P. hybrida AN2* coding sequence against genomic sequence resources: *AN2* sequences from *Nicotiana tabacum* (K326) (Sierro et al. 2014), *P. axillaris*, and *P. inflata* (Bombarely et al. 2016) were retrieved from the Sol Genomics Network website (solgenomics.net); *AN2* sequence from *Petunia exserta* was retrieved from DNA Zoo (https://www.dnazoo.org/assemblies/Petunia_exserta); and *AN2* sequence from *Petunia secreta* was retrieved from NCBI GenBank, BioProject PRJNA674325. *AN2* genomic sequences were aligned using mVista (Mayor et al. 2000) with *P. hybrida AN2* as reference, with the AVID algorithm. Detailed alignment of the *AN2-bs3* region was performed with KAlign (Lassmann 2019) and visualized with MView (Madeira et al. 2022).

### Statistical analysis

RStudio was used for statistical analysis of the numerical data. To test for differences in mean values between samples, a Shapiro–Wilk test was performed to test for normal distribution of the data, and accordingly to the results, either a Student's *t* test or a Wilcoxon rank sum test was applied. To test for differences between expected and observed frequencies, a chi-square test or a Fisher's exact test (for small samples) was applied. Details about the conditions used for the tests are given in the corresponding figure or table legends, and all statistical test results are reported in Supplemental Data Set 3.

### Accession numbers

Sequence data from this article can be found in the EMBL/GenBank data libraries under accession numbers OQ418981 (*AN1*), OQ418982 (*AN2*), and OQ418983 (*PhDEF*). Raw sequence reads for the wt, *phdef-151*, *star*, and *wico* second-whorl organ transcriptome have been deposited in BioProject with the accession number PRJNA951505.

## Supplementary Material

koad258_Supplementary_Data

## Supplemental data

The following materials are available in the online version of this article.

**Supplemental Figure S1.** Additional pictures of *star* and *wico* flowers.

**Supplemental Figure S2.** Stamens are unfused to the tube in *wico* flowers.

**Supplemental Figure S3.** Additional pictures of *PhDEF* transcript in situ hybridization in wt, *star*, and *wico* flowers.

**Supplemental Figure S4.** wt and pink wt flowers observed in the progeny of a *star* parent.

**Supplemental Figure S5.** Epidermal revertant sectors on *star* petals.

**Supplemental Figure S6.** Autonomous and nonautonomous effects in *star* and *wico* petals.

**Supplemental Figure S7.** Expression of B-class genes and a subset of pigmentation genes in wt, *star*, *wico*, and *phdef-151* samples.

**Supplemental Figure S8.** Additional information on putative PhDEF binding sites on the *AN2* genomic sequence.

**Supplemental Table S1.** Genotyping results of the progeny of a *star* flower.

**Supplemental Table S2.** List of primers used in this study.

**Supplemental Data Set 1.** Differential gene expression calculated by DESeq2.

**Supplemental Data Set 2.** List of the 451 genes downregulated in *star* and *phdef-151* samples and not differentially expressed in *wico* samples.

**Supplemental Data Set 3.** Summary of statistical analyses.

## Dive Curated Terms

The following phenotypic, genotypic, and functional terms are of significance to the work described in this paper:

PIN1 Gramene: AT1G73590
PIN1 Araport: AT1G73590

DFR Gramene: Peaxi162Scf00366g00630
DFR Araport: Peaxi162Scf00366g00630

AP3 Gramene: AT3G54340
AP3 Araport: AT3G54340

JAF13 Gramene: Peaxi162Scf00119g00942
JAF13 Araport: Peaxi162Scf00119g00942

DPL Gramene: Peaxi162Scf01210g00002
DPL Araport: Peaxi162Scf01210g00002

BRI1 Gramene: AT4G39400
BRI1 Araport: AT4G39400

NEK6 Gramene: AT3G44200
NEK6 Araport: AT3G44200
