# Supplementary material for: Cell layer–specific expression of the homeotic MADS-box transcription factor PhDEF contributes to modular petal morphogenesis in petunia
Source: Plant Cell. 2023 Oct 6;36(2):324–45. doi: 10.1093/plcell/koad258 (PMC10827313; doi:10.1093/plcell/koad258)
Supplement: koad258_Supplementary_Data [file koad258_supplementary_data.zip › tpc.23.00287Supplemental Figures and Tables.pdf]

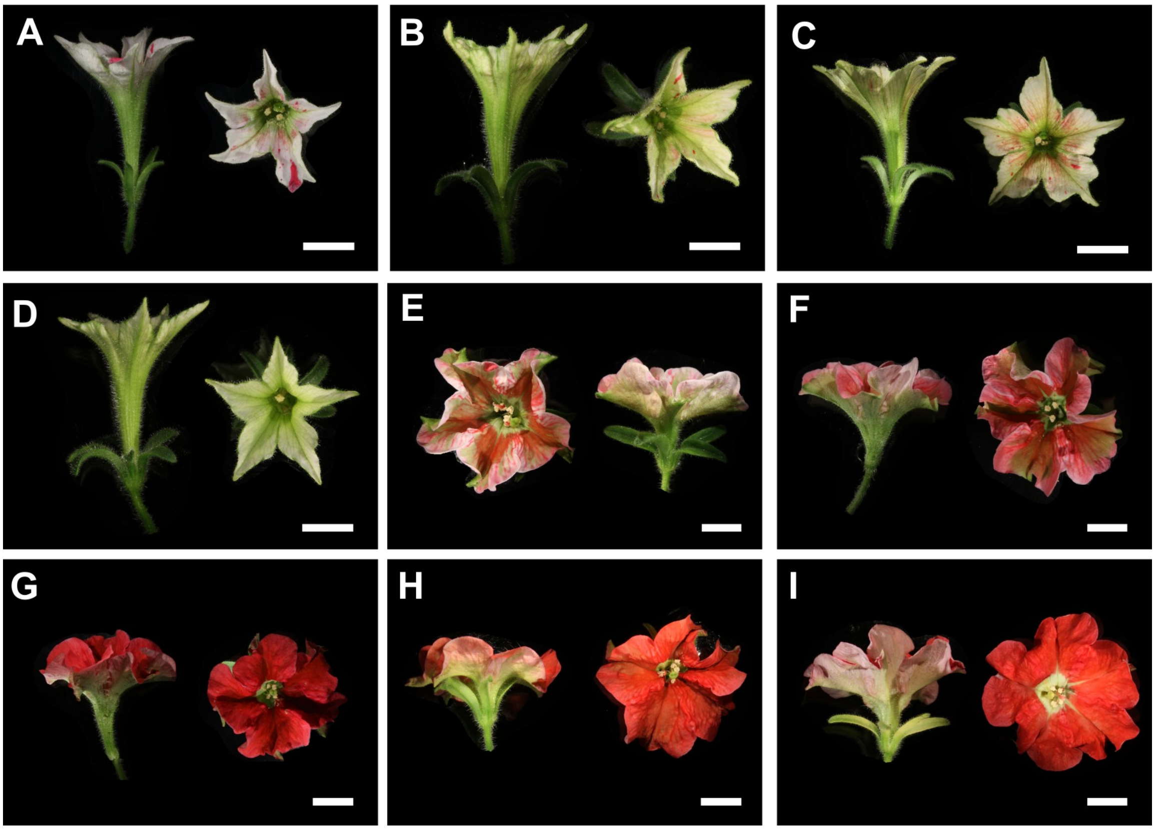

Supplemental Figure S1. Additional pictures of *star* and *wico* flowers (Supports Figure 1). *Star* (A-D) and *wico* (E-I) flowers from independent branches, viewed from the side (left) and from the top (right). Sepals have been occasionally removed for clarity. Scale bars: 1 cm.

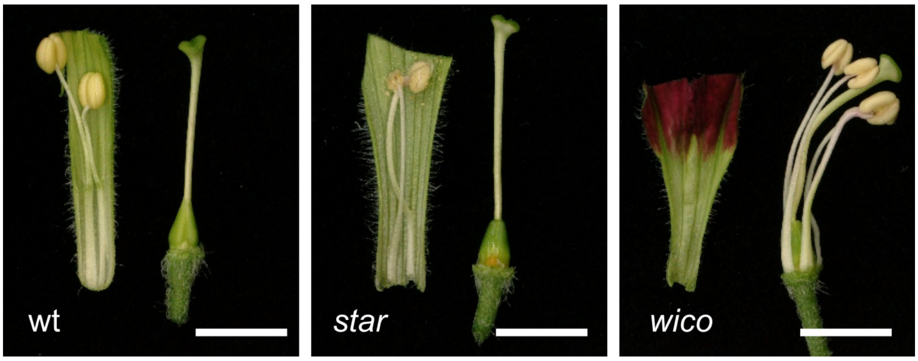

Supplemental Figure S2. Stamens are unfused to the tube in *wico* flowers (Supports Figure 1).
The base of the corolla (left) was detached from the gynoecium (right) in wild-type (wt), *star* and *wico* flowers. In wt and *star* flowers, the stamens are fused to the petal tube at their base. In *wico* flowers, the stamens are unfused to the petal tube and therefore remain attached to the flower receptacle. Scale bars: 0.5 cm.

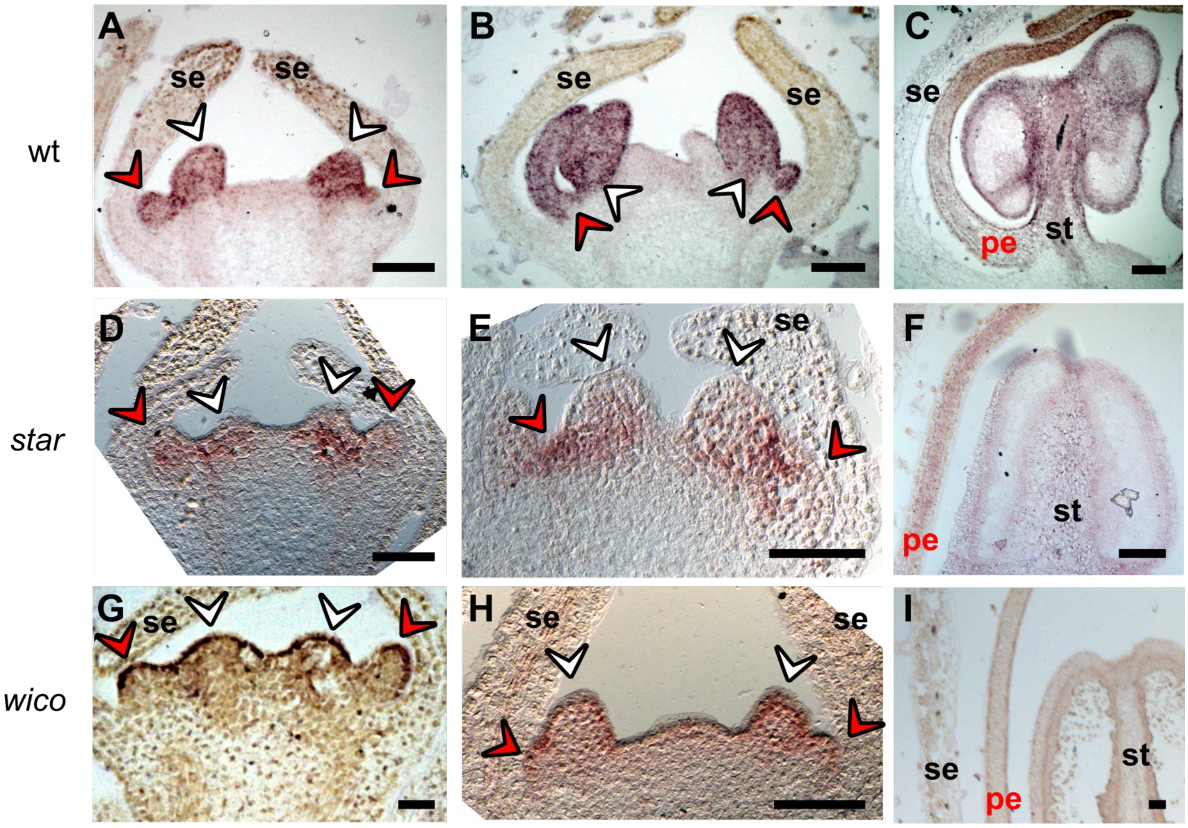

Supplemental Figure S3. Additional pictures of *PhDEF* transcript *in situ* hybridization in wild-type, *star* and *wico* flowers (Supports Figure 3).
Longitudinal sections of wild-type (wt) (A-C), *star* (D-F) and *wico* (G-I) flowers at various stages (increasing stages from left to right) hybridized with a digoxigenin-labelled *PhDEF* antisense probe. Red and white arrowheads indicate initiating petals and stamens respectively. se: sepals, pe: petals, st: stamen. Scale bar: 20 µm (A, B, D, E, G, H) or 50 µm (C, F, I).

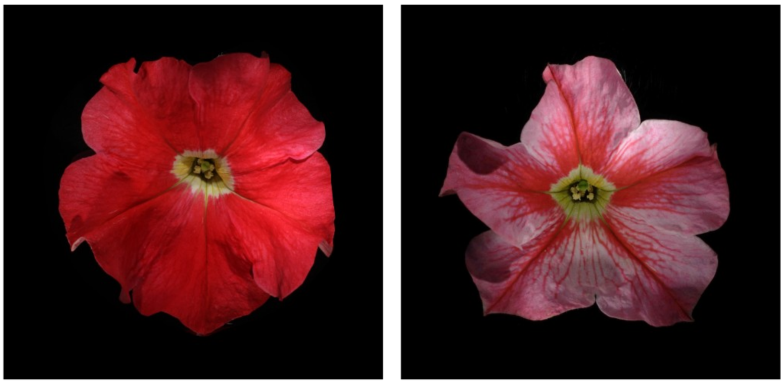

Supplemental Figure S4. Wild-type and pink wild-type flowers observed in the progeny of a *star* parent (Supports Table 1).
Pink wild-type flowers (right) carry a *PhDEF+6* allele and an out-of-frame *phdef* allele, whereas flowers carrying *PhDEF+6* alleles at the homozygous state appear wild-type (left).

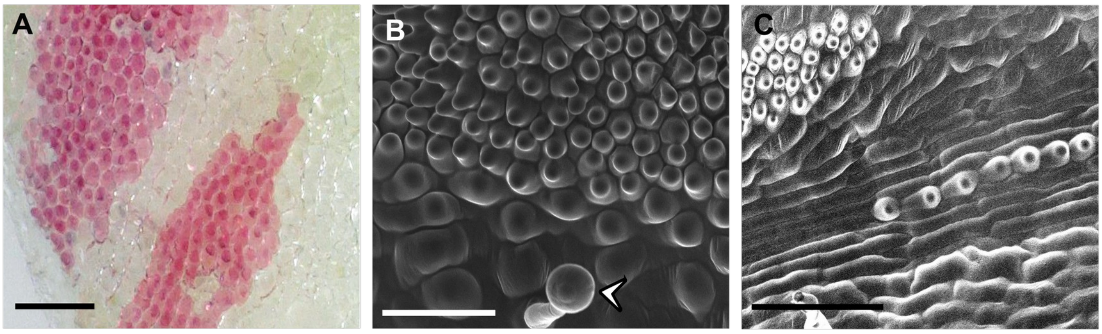

Supplemental Figure S5. Epidermal revertant sectors on *star* petals (Supports Figure 4). **(A)** Detail of a small pink revertant sector found on a *star* flower, showing a sharp transition between pigmented and non-pigmented epidermal cells. Scale bar: 100 µm. **(B)** Scanning electron micrograph of the boundary between *star* and pink revertant petal sectors, showing a quite sharp transition in conical cell shape and size. The white arrow indicates a trichome. Scale bar = 50 µm. **(C)** Scanning electron micrograph of a file of pigmented revertant cells on a *star* petal limb, located along a vein, showing a sharp transition in conical cell shape. Scale bar: 100 µm.

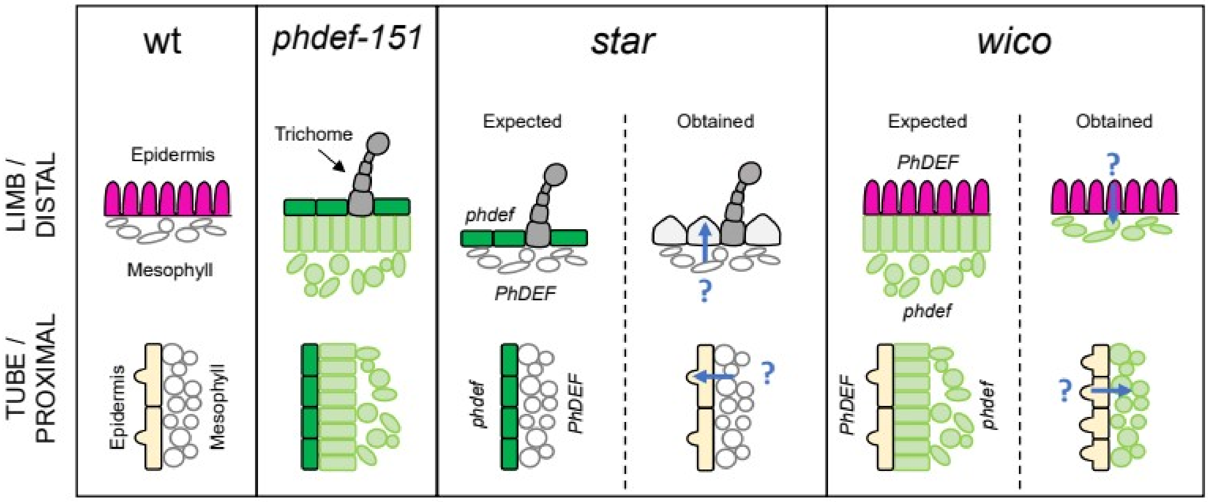

Supplemental Figure S6. Autonomous and non-autonomous effects in *star* and *wico* petals (Supports Figure 4).
Schematic cross-sections of the second whorl organs from wild-type (wt), *phdef-151*, *wico* and *star* flowers, for the distal (limb for petals) and proximal (tube for petals) parts of the organs. Adaxial epidermal cells and mesophyll layer cells are schematized based on cross-sections from Fig. 4, and abaxial epidermal cells have been omitted. For *phdef-151*, the mesophyll is divided in palisade and spongy layers. For *wico* and *star*, we propose a schematic view of the expected cross-sections, based on the genotype of the flower, and of the cross-sections that we obtained. The difference between the two suggests the existence of non-autonomous effects (blue arrow) of an unknown nature.

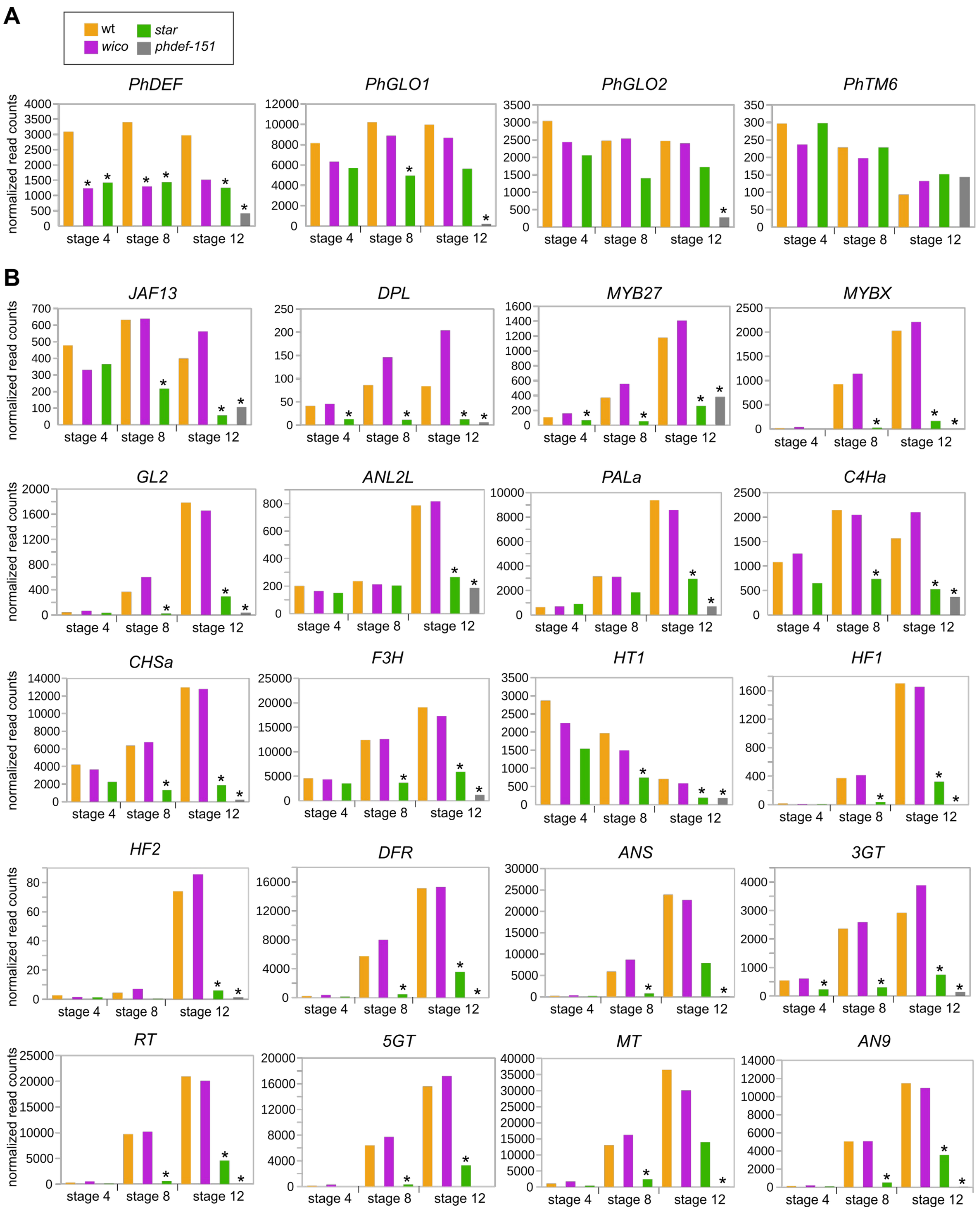

Supplemental Figure S7. Expression of B-class genes and a subset of pigmentation genes in wild-type, *star*, *wico* and *phdef-151* samples (Supports Figure 5).
Expression (as normalized read counts calculated by DESeq2) of B-class genes (A) and a subset of pigmentation genes (B) in wild-type (wt), *star*, *wico* and *phdef-151* second whorl organs at stages 4, 8 or 12. The subset of pigmentation genes are the 23 genes

significantly down-regulated in *star* samples (at any stage) and *phdef-151* samples, and not differentially expressed in *wico* petals, as in Supplemental Dataset S2. Stars indicate significant down-regulation ($log_2FC < -1$ and adjusted p-value < 0.01).

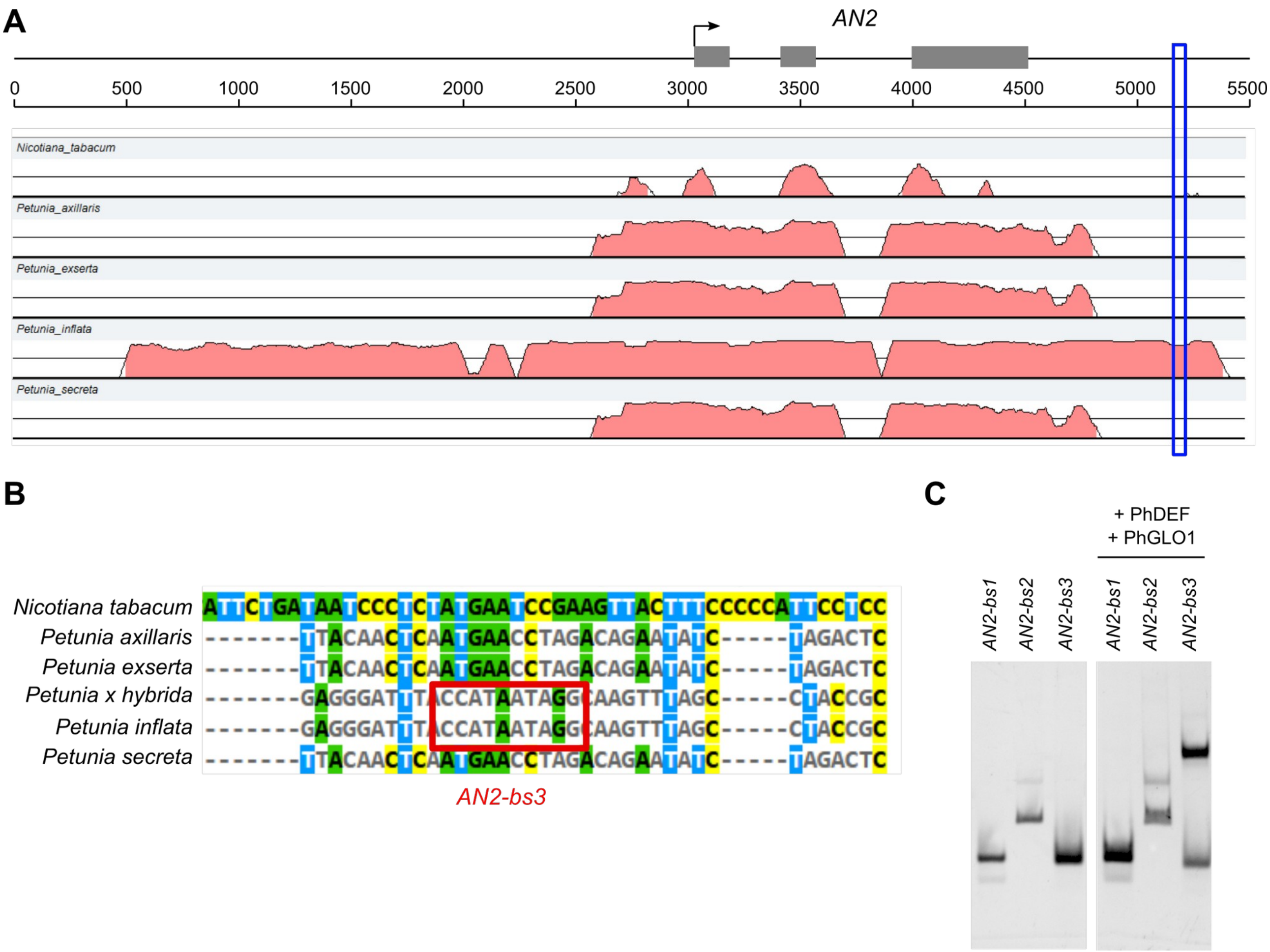

Supplemental Figure S8. Additional information on putative PhDEF binding sites on the *AN2* genomic sequence (Supports Figure 6).
(A) Conservation plot of a pair-wise alignment of *P. axillaris*, *P. exserta*, *P. inflata*, *P. secreta* and *N. tabacum AN2* sequences to *P. hybrida AN2* sequence. The y-axis shows the percentage of conservation of the sequence (all regions above 50% conservation are displayed, all regions above 70% conservation are colored in pink). The region where *AN2-bs3* is found is shown with a blue rectangle. The gene model for *AN2* is shown above the conservation plot (exons as grey rectangles).
(B) Multiple sequence alignment of the region around *AN2-bs3* in *P. axillaris*, *P. exserta*, *P. inflata*, *P. secreta, P. hybrida* and *N. tabacum AN2* sequences. The exact *AN2-bs3* CArG box is shown with a red rectangle.
(C) Electrophoretic mobility shift assay (EMSA) gel performed with a combination of *in vitro*-translated PhDEF and/or PhGLO1 proteins, and Cy5-labelled *AN2-bs1, AN2-bs2* or *AN2-bs3* DNA fragments, whose position is depicted in main Figure 6. *AN2-bs2* is larger than other DNA fragments and could not be extremely well purified, therefore two bands are present in the DNA fragment alone.

Plant	Phenotype	Genotype
1	wt	*PhDEF+6 / PhDEF+6*
2	wt	*PhDEF+6 / PhDEF+6*
3	wt	*PhDEF+6 / PhDEF+6*
4	wt	*PhDEF+6 / PhDEF+6*
5	pink wt	*phdef-151 / PhDEF+6*
6	pink wt	*phdef-151 / PhDEF+6*
7	pink wt	*phdef-151 / PhDEF+6*
8	pink wt	*phdef-151 / PhDEF+6*
9	pink wt	*phdef-151 / PhDEF+6*
10	pink wt	*PhDEF+7 / PhDEF+6*
11	pink wt	*phdef-151 / PhDEF+6*

Supplemental Table S1. Genotyping results of the progeny of a *star* flower (Supports Table 1).
11 plants with flowers with a wild-type flower architecture, descendant from a single *star* flower after selfing, were classified into wild-type (wt) or pink wt phenotypes (see Fig. S3). These plants were genotyped and sequenced at the *PhDEF* locus. All plants with flowers with a wt appearance carried in-frame *PhDEF+6* alleles at the homozygous state. All plants with flowers with a pink wt appearance carried an out-of-frame *PhDEF* allele (*phdef-151* or *PhDEF+7* allele) and an in-frame *PhDEF+6* allele.

Target gene	Application	Primers	5'-3' sequence
PhDEF	Genotyping *phdef-151*	MLY0935<br>MLY0936	GAACTCACTGTTCTTTGTGATGC<br>GAGAATACTAGTTCTTGGATACGTAC
	In situ hybridization	MLY1738<br>MLY1739	GACTAAGCAGTTGTTCGATCTGTAC<br>tgtaatacgactcactatagggcTACTCAAGCAGAGCAAAAGTAGTG
	Cloning into pSPUTK	MLY2382<br>MLY2383	AACATGCCATGGCTCGTGGAAAGATCCAGA<br>AACATGTCTAGACTACTCAAGCAGAGCAAAAGTAGT
	ChIP (PhDEF^{GF1})	MLY2626<br>MLY2627	GCAACACCTTTCACGGTTTTGGCAA<br>CTTGTAGATTTAGAGATAAGATTCTGGAGG
PhGLO1	Cloning into pSPUTK	MLY2384<br>MLY2385	AACATGCCATGGGGAGAGGAAAGATAGAGATAAA<br>AACATGTCTAGATTACAACCTCTCCTGCAAATTTGG
AN1	terminator cloning	MLY2334<br>MLY2335	CAAGAAGTCAATACACCAGTTAATCCC<br>GCCATCTATTGTTGCATTAGCG
	AN1-bs1 amplification	MLY2336<br>MLY2337	Cy5-TTTATCCCAAAATAAGTGCCTC<br>AACAAATTTTTGAATTAAAGAGTTAC
	AN1-bs2 amplification	MLY2338<br>MLY2339	Cy5-TTCACAAACATCAAAGTTTAGC<br>CTAGGGAATAGAGCAAGTAGC
	ChIP (AN1^{GF1})	MLY2643<br>MLY2644	CAAGAATCAATCGTTGTATTAGTCTCTGTCAAAT<br>GCGAATTCCACGAAAAGCGGTGTG
	ChIP (AN1^{GF2})	MLY2630<br>MLY2631	TGTCGACCATTCTTGAACACCTCTCAAAC<br>GGTGCTGGGGCTGGGCCACC
	ChIP (AN1^{GF3})	MLY2641<br>MLY2642	CCTCTGTTCCATAATGAGTGTCTATCTTTTC<br>CCTTTGGTCTAAAGCTAAAAGTGTGTGTAAT
AN2	terminator cloning	MLY2440<br>MLY2441	GGGATTTACTTGGTTAATTGGGACC<br>CCAAACATTAAAATCTCTCCAAACTAA
	promoter cloning	MLY2442<br>MLY2443	ACCCCATAAGAAATAAATGGACCCTATC<br>ATTGAATCGATGGTACATCTAAAACTATG
	AN2-bs1 amplification	MLY2446<br>MLY2447	Cy5-GATGAATGAGTCCCCTATTTATA<br>TACTTGGTGCCCAAGATAGG
	AN2-bs2 amplification	MLY2448<br>MLY2449	Cy5-GGGACCATTGAAGAGATTAAGA<br>CAAATGTCCAACGATTTCAACT
	AN2-bs3 amplification	MLY2450<br>MLY2451	Cy5-TTATGTACGGTTGTGAGGGA<br>AGGAGAGTTGCGGTAGGC
	ChIP (AN2^{GF1})	MLY2737<br>MLY2738	GGAATGAACTCATAGTTTTAGATGTACCATCG<br>GCCAAACTTGCATGAAAATTACCACAACTAT
	ChIP (AN2^{GF2})	MLY2636<br>MLY2637	CTTCATAAGCTTCTAGGCAACAGGTAAG<br>GAGATGCTAAAGGGGAAGTTCAGGGTT
	ChIP (AN2^{GF3})	MLY2634<br>MLY2635	CCACATAGCAACATATGTAACCCTTTCTTTTT<br>CGATCCCAAATCGAAATAAGTGAGGAG
Neg1 (Peaxi162Scf00207g00634)	ChIP	MLY2645<br>MLY2646	GGAGACATAGGATTCACATATCCAACCCAAC<br>GGAGAAATTAAACACTTCCCCACTAGTTGATTA
Neg2 (Peaxi162Scf00164g00525)	ChIP	MLY2647<br>MLY2648	CGGCATTCATGGATTTCTTATATTGACCTG<br>TCTAGAAATGACATGAAAAACCATTAGGAGTACT

Supplemental Table S2. List of primers used in this study.
For MLY1739, the T7 Polymerase promoter sequence is in red. For MLY2382 to MLY2385, the XbaI or NcoI restriction sites are in red.
