# Supplementary material for: Cell layer–specific expression of the homeotic MADS-box transcription factor PhDEF contributes to modular petal morphogenesis in petunia
Source: Plant Cell. 2023 Oct 6;36(2):324–45. doi: 10.1093/plcell/koad258 (PMC10827313; doi:10.1093/plcell/koad258)
Supplement: koad258_Supplementary_Data [file tpc23.00287Peer Review Report.pdf › tpc23.00287Peer Review Report.pdf]

Cell layer-specific expression of the homeotic MADS-box transcription factor PhDEF contributes to modular petunia petal morphogenesis

Chopy M., Cavallini-Speisser Q., Chambrier P., Morel P., Just J., Hugouvieux V., Rodrigues Bento S., Zubieta C., Vandenbussche M., and Monniaux M.

Corresponding author: Vandenbussche M (michiel.vandenbussche@ens-lyon.fr) and Monniaux M (marie.monniaux@ens-lyon.fr)

Review timeline:

**TPC2023-RA-00287**	Submission received:	March 27, 2023
	1st Decision:	May 19, 2023 *revision requested*
**TPC2023-RA-00287R1**	1st Revision received:	August 30, 2023
	2nd Decision:	September 17, 2023 *accept*
	Final acceptance:	October 3, 2023

REPORT: (The report shows the major requests for revision and author responses. Minor comments for revision and miscellaneous correspondence are not included. The original format may not be reflected in this compilation, but the reviewer comments and author responses are not edited, except to correct minor typographical or spelling errors that could be a source of ambiguity.)

TPC2023-RA-00287 1st Editorial decision – *revision requested* **May 19, 2023**

We have received reviews of your manuscript entitled "Cell layer-specific expression of the homeotic MADS-box transcription factor PhDEF contributes to modular petunia petal morphogenesis." Thank you for submitting your best work to The Plant Cell. The editorial board agrees that the work you describe is substantive, falls within the scope of the journal, and may become acceptable for publication, pending revision and potential re-review.

In preparing your revision, please pay special attention to (1) the comments from all reviewers on further improving the clarity and readability of the manuscript, and (2) the comments from reviewers one and three regarding PhDEF binding sites/CArG boxes. Please contact us if there are ambiguous comments or if you wish to discuss the revision.

Given the nature of the comments, we are offering you 60 days from when we have issued this decision to complete the revision. If a revision is not returned within this time frame, and if you have not been granted an extension, we will withdraw the manuscript, which will leave you free to submit the work elsewhere. If you need an extension, we encourage you to contact us at any point before the 60 days have passed.

--- Reviewer comments:

TPC2023-RA-00287R1 1st Revision received **August 30, 2023**

Reviewer comments and **author responses**:

Reviewer #1:

This study describes the function of B class MADS-box gene PhDEF in petunia petal development from two aspects: the modular development of the limb and tube, and the regulation of pigmentation genes. First, the authors focused on two types of spontaneous morphological alterations that took place in phdef-151, a loss of function allele due to a transposon insertion. One of them is "star", which produced relatively normal tubes with reduced limbs. Another is "wico", which had highly reduced tubes with relatively normal limbs with pigmentation. Molecular genetic analysis and in situ hybridizations revealed that star and wico are periclinal chimeras of revertants to the wild type in the L1 and L2 layers, respectively, due to the transposon excision from phdef-151, suggesting that formation of the limb and the tube are regulated in a modular manner driven by the epidermal and subepidermal PhDEF functions. Morphological and histological observations in these revertants also suggested the presence of non-cell autonomous effects on the differentiation of L1 and L2 layers in petunia petals. In the second half of this study, the authors performed transcriptome analysis and identified sets of genes differentially expressed in these mutants. They focused on the anthocyanin synthesis pathway and showed that PhDEF and its cofactor PhGLO1/2 directly bind to the AN2 locus, a key transcription factor in this pathway.

The description of star and wico in the first half is beautiful; it nicely showed the modular contribution of L1 and L2 layers

through PhDEF to tube and limb formation. This will be significant to understand the petal development in petunia, which is of great importance in targeting different pollinators. The second part is also interesting because it suggests a direct link between a floral homeotic regulator and petal pigmentation. Although these two sections are both interesting and valuable, my concern is that the association between these two parts is not very strong; they may be recognized as different topics. In addition, the second part about the functional analysis of PhDEF in the regulation of AN2 may need more validation.

<Major concerns>

・My understanding is that the authors focused on the pigmentation pathway as an example of the modular regulation found in the first half. If this is correct, I think the authors should explain more carefully why they focused on the pigmentation pathway. This can also be applicable to the abstract.

We agree that the two sections of the study might be viewed as two different topics. However, we believe that associating them together provides a proof-of-concept of how the *phdef* periclinal chimeras can be used to characterize the link between petal identity and layer-specific phenotypes, such as pigmentation. The main layer-specific phenotypes controlled by PhDEF that our chimeras revealed are limb growth, conical cell formation and pigmentation (from PhDEF activity in the epidermis) and tube growth (from PhDEF activity in the mesophyll). The reasons for focusing on pigmentation are the following:

genes involved in growth of the limb or the tube are not identified yet and might be numerous, therefore this process was not straightforward to investigate further from our transcriptomic dataset;

similarly, to date only *PhMYB1* (from the *MIXTA* gene family) has been clearly identified to regulate conical cell formation in the petunia petal epidermis. The full gene network controlling this trait still remains to be established, therefore it did not appear to us as the most straightforward choice;

as mentioned in the manuscript, the anthocyanin production pathway is extremely well described in petunia. The most upstream regulators of anthocyanin production have been well characterized, together with their mutant phenotypes, which gave us a solid framework to test the link with PhDEF activity. It is an ideal situation for the proof-of-concept that we were trying to establish.

We have better explained our rationale in the Abstract and in the Results section (lines 370-381).

・I think that star and wico phenotypes can also be explained by cell lineages in case a large part of the limb is derived from the L1 layer. In this case, star phenotype is simply explained by a lack of cells that serve as the source of the limb with a correct identity. Similarly, the wico phenotype can be explained by the recovery of the cells with petal identity that generate the limb. Thus, if the limb is derived largely from the L1 layer, the nature of this modular regulation of petal development can be explained by the cell lineages.

Thank you very much for this remark. We fully agree with your viewpoint. In the previous version of the manuscript, we mentioned already in the last phrase of the abstract « Altogether, we show that cell-layer specific floral homeotic activity in petunia petals differently impacts tube and limb development, revealing the relative importance of the different cell layers in the modular architecture of petunia petals. », and we discussed this in more details in the paragraph

« Contribution of cell layers to mature petunia petals » in the Discussion section, although it indeed could have been more clearly stated. We have now modified the Discussion to better integrate this idea (lines 503-508).

・Although overall growth of the tube is recovered close to the wild type in star tubes, its inner tissue organization is not the same as that in the wild type (Fig 4C). I think this indicates that PhDEF function in the L2 layer is not sufficient for the development of tube mesophylls.

Indeed, the mesophyll organization in the tube star flowers appears different to wild-type, with bigger cells and less lacunae between cells. We have checked on other star tube sections and the section in Fig. 4C is a representative one for this observation. This indeed suggests that PhDEF activity in the L2 layer is not entirely sufficient for petal mesophyll formation in the tube. Still, the overall organization of the tissue appears more similar to a petal mesophyll than to a sepal mesophyll, since there is no cellular differentiation into a palisade and spongy layer. We have nuanced our interpretation of Fig. 4C in the Results section, lines 311-314.

・Experiments provided here showed the direct binding of PhDEF to AN2 locus, however, structural and functional aspects of this binding are not examined. First, the exact nucleotide sequence of the PhDEF binding site is not provided. Is this site conserved among related species? In addition, is it possible to test how AN2 expression is affected when the binding site is mutated?

We have now provided additional details on the PhDEF binding site (*AN2-bs3*) that we have identified in the AN2 terminator region. Its sequence corresponds to a consensus CArG box: CCATAATAGG (sequences of *AN1-bs1* and *AN2-bs3* added lines 424-426). It lies relatively far from the coding region (744 bp downstream of the STOP codon) and this region is overall not conserved between closely-related species (see alignment in Supplemental Figure 8). We have found the *AN2-bs3* site to be present in *Petunia inflata*, one of the wild parents from *Petunia x hybrida*, and in which *AN2* triggers the

accumulation of anthocyanin in the pink pigmented petals. *AN2-bs3* is not present in *P. axillaris* (the other wild parent from *P. hybrida*) that lost anthocyanin accumulation in the petal due to a frame-shift mutation in *AN2* (Quattrocchio et al., 1999). *AN2-bs3* is also not found in *P. exserta* (regain of petal pigmentation, but controlled by *DPL* rather than *AN2*, Berardi et al., 2021), nor in *P. secreta* (regain of petal pigmentation by restoration of the reading frame in *AN2*, Esfeld et al., 2018). Finally, we could not find *AN2-bs3* in *Nicotiana tabacum AN2*, that also controls anthocyanin deposition in the petals (Pattanaik et al., 2010). Sequence alignment of *AN2-bs3* can be found in Supplemental Figure 8, and score predictions for MADS-box proteins on the *AN2* genomic sequence from wild Petunia species can be found in the figure below. It is to be noted that another putative PhDEF binding site (noted here *AN2-bs4*), lying in the second intron of *AN2*, is conserved between *P. inflata, P. axillaris, P. exserta* and *P. secreta* (high score and conserved sequence), but this site is not present in *P. hybrida*. This putative binding site might play a role in *AN2* regulation by PhDEF in wild *Petunia* species, but this remains to be tested and we have not included this information in the revised version of our manuscript.

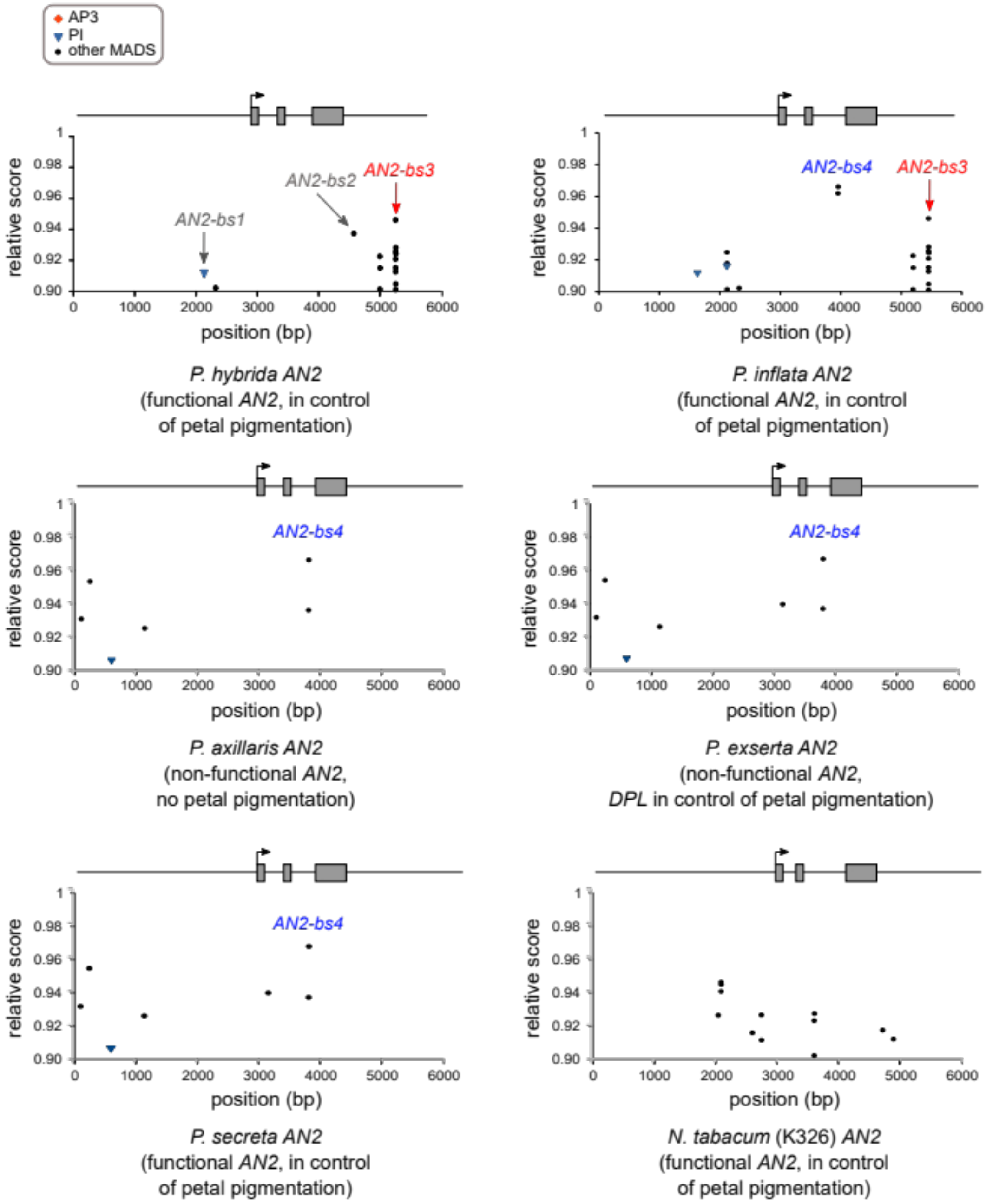

Petal pigmentation is an extremely labile trait. Even within the *Petunia* genus, the role of *AN2* in controlling petal pigmentation has changed because petal pigmentation has been lost and regained, and in *P. exserta* the function of *AN2* has been coopted by its close paralog *DPL*. Indeed, *AN2* is part of the very large R2R3-MYB family, and unless thorough functional experiments are undertaken in a given species, it is unclear which member(s) of the family control petal pigmentation.

Therefore, we could not extend our analysis beyond *Petunia* and *Nicotiana* and, to our opinion, the fact that *AN2-bs3* is not conserved is not necessarily an argument for its lack of biological relevance, since it might be a recently evolved binding site for PhDEF.

We have added these arguments in the revised version of the manuscript (lines 628-636 in the Discussion).

Finally, we have attempted to test the function of *AN2-bs3* in the putative regulation of *AN2* by PhDEF, as suggested by the reviewer. For this, we have performed a dual luciferase assay in *Petunia hybrida* mature petals (where *PhDEF, PhGLO1* and *SEPALLATAs* are expressed at strong levels), by infiltrating different synthetic reporters:

the positive control *pPhDEF::luciferase:tCaMV* (1.2 kb *PhDEF* promoter controlling the expression of the firefly luciferase, with the standard cauliflower mosaic virus terminator);

the test construct *pAN2::luciferase:tAN2* (1.4 kb *AN2* promoter controlling the expressing of the firefly luciferase, followed by 1 kb of *AN2* terminator region containing the *AN2-bs3* site);

the test construct *pAN2::luciferase:tAN2m* (1.4 kb *AN2* promoter controlling the expressing of the firefly luciferase, followed by 1 kb of *AN2* terminator region containing the mutated *AN2-bs3* site). The mutation of *AN2-bs3* that we performed (CCATAATAGG mutated to TTATAATAAA) is expected to fully disrupt MADS-box protein binding (Jetha et al., Nucleic Acid Research 2014).

Our results are shown in the following figure (8 biological replicates and 3 technical replicates for each).

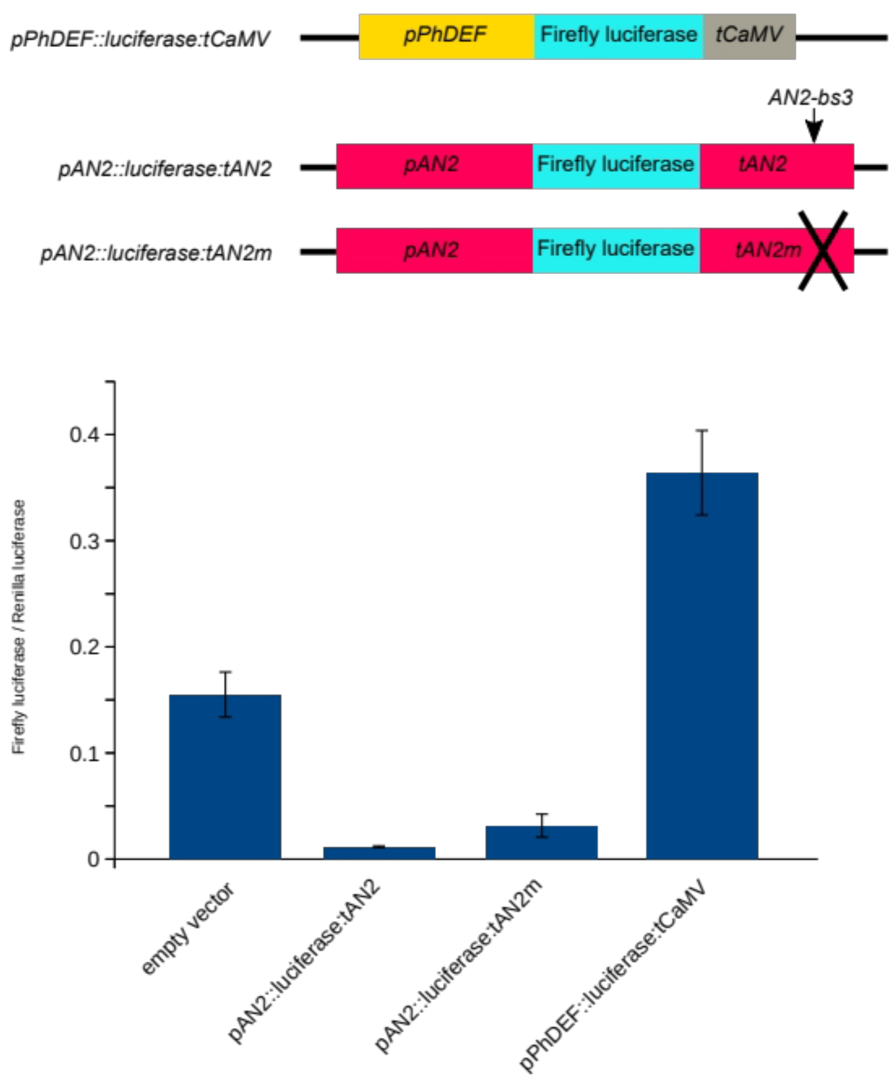

Our positive control with the *PhDEF* promoter shows that the luciferase assay is working in our hands with a strong expression of the Firefly luciferase, as expected since *PhDEF* activates its own expression. However, our test construct *pAN2::luciferase:tAN2* shows a much weaker expression of the luciferase, even weaker than the one of the empty vector. From our RNA-Seq data, we know that endogenous expression levels of *PhDEF* and *AN2* are similar in close-to-mature petals (2970 normalized reads for *PhDEF* vs. 2355 normalized reads for *AN2*, at stage 12 just before flower opening). Therefore, the fact that we see a ca. 80-fold lower expression of luciferase with the *AN2* construct than with the *PhDEF*

construct suggests that our *AN2* synthetic construct is not working properly. Indeed, since *AN2-bs3* is located in the terminator region of *AN2*, *AN2* regulation by PhDEF might relie on a complex genomic context that we are unable to recreate in a simple synthetic construct. When mutating *AN2-bs3* in this context, we did not detect any significant change in luciferase expression, but we consider this result to be meaningless.

Due to these limitations that prevent us from drawing any conclusions, we have decided not to include these results in the revised version of the manuscript. However, since we understand that we are unable to provide any strong argument for the functionality of *AN2-bs3* in regulating *AN2* expression, we have tempered our conclusions on this point throughout the manuscript. In particular lines 32-33 of the Abstract, line 130 of the Introduction, lines 403-404 of the Results section and lines 643-644 of the Discussion section.

- The title of this manuscript does not mention the second part. How about adding "and petal pigmentation" at the end?

Since we were unable to provide functional support for PhDEF regulating *AN2* expression through the binding to *AN2-bs3*, we decided not to add « petal pigmentation » at the end of the title.

<Minor comments>

- In the abstract, the words "periclinal chimera" might be better than "mutants" to transmit the

message of this study to readers.

To our knowledge, most readers will not be familiar with the term « periclinal chimeras » and therefore might not understand the abstract on a first reading, if only this term is present. We have therefore decided to keep the word « mutant » with a description of the layer-specific *PhDEF* expression, and added « (periclinal chimeras) » for the experienced readers.

- In Figure 1K, please indicate the limb region so that readers unfamiliar with petunia terminology can understand the petal structure.

We have indicated the tube and the limb regions in Fig. 1B and Fig. 1K.

- In Supplementary Figure 6, please switch the order of panels of wico and star because it will be helpful for readers reading Figure 4. Currently, the orders of mutants in these two figures are different.

Thank you for this suggestion, we have inverted the order of star and wico panels in Supplementary Figure 6.

Reviewer #2:

In their manuscript entitled "Cell layer-specific expression of the homeotic MADS-box transcription factor PhDEF contributes to modular petunia petal morphogenesis", Chopy et al. describe two novel phenotypes derived from a def transposon mutant, whereby either petal tubes or lobes are affected. These novel mutant flowers show excision of the transposon, either resulting in wild type DEF or a DEF protein with two additional amino acids. The wico and star mutants are differentiated by whether the reverted (or semi-reverted) DEF occurs in the L1 or L2 layer, as evidenced by the progeny genotypes derived from L2. The wico and star phenotypes are caused by cell autonomous and non-cell-autonomous effects of DEF, and DEF binds directly with at least one anthocyanin pathway gene, as inferred from RNAseq, a gel shift assay, and ChIP. This work is a very interesting addition to what we know about the function of MADS-box homeotic genes in floral organ development. Although similar mutants have been characterized in snapdragon, and cell layer-specific expression has been done in A. thaliana, I still think this study in petunia is valuable. It is also very well presented and logical.

Thank you for these positive comments. Thank you also for noticing typos throughout the manuscript and for suggesting changes to improve its readibility.

I only have minor suggestions:

Line 55: there is an 'a' that needs to be in italics.

This has been corrected.

Line 145-148: there is no figure or paper cited for this description of the dTph1 insertion def mutant. I see a representative flower in Fig. 1C, but shouldn't there be some statistics done on this? Or at least a mention of how many flowers were surveyed.

We have grown the *phdef-151* mutant for several years in our laboratory, and we have observed hundreds of flowers (which we have now mentioned in the Methods section of the manuscript, line 658-660). All of them display the same phenotype, for which the flower in Fig. 1C is a representative example. As already mentioned in the manuscript, the phenotype is also identical to the one of other *phdef* mutant alleles previously reported (*def-1* or *green petal* (*gp*) from de Vlaming 1984 and van der Krol 1993).

Line 157: please make sure to indicate that 'wt' is an abbreviation for wild-type.

This was indicated in the Introduction (line 126) but we have now also indicated in the Results section (line 157).

Line 170: spell check simultaneous and restoration.

This has been corrected.

Line 298: replace "than wt cells" with "to wt cells".

This has been corrected.

Line 355: I wonder if a better term could be used to replace "deregulated". This implies that these genes are not regulated at all, which of course cannot be the case. Perhaps "misregulated" or simply just differentially expressed genes (DEGs).

Thank you for this comment, indeed « deregulated » is not an appropriate term. We have modified « deregulated genes » by « differentially expressed genes » or DEGs throughout the manuscript, and mainly in lines 342-355 of the revised version.

Line 386: 'to whom' should be corrected to 'with whom'.

This has been corrected.

Line 393: remove 'a' from 'a particular attention'.

This has been corrected.

Line 478: I think Datura needs to be in italics, but maybe this is a TPC specific thing.

It is now in italics and we have specified the species name (*Datura stramonium*).

Line 481: change 'participates to...' to 'participates in forming the vaculature'.

This has been corrected throughout the manuscript.

Line 496: instead of 'as we progress', perhaps 'progressing from'?

This has been corrected.

Line 530: change 'participate to' to 'participate in'.

This has been corrected throughout the manuscript.

Line 555: this is a giant paragraph. I'd suggest splitting it into two.

Thank you for this suggestion, we have now splitted this paragraph into three sections (summary of the autonomous and non-autonomous effects observed / discussion about PhDEF movement between layers / discussion about mechanical effects).

Line 590-590: I'd suggest 'one the one hand...on the other.'. This has been modified.

Line 595: replace 'it is not obvious' to 'it is not easy'. This has been modified.

Line 596: perhaps single cell RNAseq instead of 'spatial transcriptomics'?

We have modified as follows, lines 599-601: « Spatial transcriptomics techniques, such as single- cell RNA-Seq, would be ideal... », because to be perfectly correct, other techniques (Slide-Seq for instance) would be adapted to our question as well.

Line 625: replace 'thereby preventing to draw' with 'thereby preventing us to draw'.

This has been corrected.

Line 628: I think this should say 'fill in the missing link'.

This has been corrected.

Line 634: it would be nice for other petunia folk if you can say briefly how you propagated the cuttings (e.g rooting media?).

We have added details on how cuttings are performed in the Methods section (lines 661-665), although this is a procedure that is widely-spread in the petunia community.

Line 684: 'grounded' should be 'ground'.

This has been corrected.

Figure 1A: it would be useful to indicate which branch is the wico and which the star mutant.

We have now indicated the star and wico branches in Figure 1A.

Figures 3 and 4: even though it is stated in the text, I think it would be very useful to indicate the epidermal and mesophyll layers in the two figures. This allows the potentially naïve reader to easily understand the relationship between L1-epidermis and L2-mesophyll and the concept of cell autonomous and cell-non-autonomous.

We have now added in the wild-type samples the L1/L2/L3 layers (in the floral meristem) and epidermis/mesophyll layers (in the petal) in Figures 3 and 4.

Reviewer #3:

In the manuscript `Cell layer-specific expression of the homeotic MADS-box transcription factor PhDEF contributes to modular petunia petal morphogenesis', Chopy et al. identified two types of transposon mutant flowers for the PhDEF gene, which display cell layer-specific revertance to the wild-type phenotype. Interestingly, both mutants display a different phenotype, dependent on the cell layer that has become wild-type again. In the wico mutant flowers, the L2 layer is still homozygous mutant, but the transposable element in the L1 layer excised in one allele, restoring a functional PhDEF allele in the L1 layer. In star mutant flowers, the opposite has occurred, restoring PhDEF function in the L2 layer. In Petunia, loss of PhDEF results in the conversion of petals into sepals, and the layer-specific restoration of PhDEF function gives a unique insight into how PhDEF regulates the specific formation of petal tissue and in which cell layer the different aspects of petal development are mainly regulated. Interestingly, PhDEF expression in the mesophyll (L2) is mainly required to ensure the formation of the long petal tube, while its expression in the epidermis (L1) is essential to form the colourful petal-limb. The authors also show that the pigmentation of the limb is regulated by PhDEF in the epidermis via direct binding to AN2. The interesting findings are partially novel and partially show the conservation/divergence of cell-layer specific regulation in petal development compared to Datura, Antirrhinum and Arabidopsis.

Comments:

- The manuscript is well-written, though sometimes a bit more concise explanation of the results would make them clearer.

Based on this comment and remarks from the other reviewers, we have attempted to clarify and simplify the manuscript at various places. In particular, we have simplified the Results section about autonomous and non-autonomous effects to make it more concise.

The authors write star and wico in regular font, but writing them in italics would have my preference (to distinguish them easier from the rest of the text).

We agree that since they are partial mutants, it is more logical to write their name in italics. This has been modified throughout the manuscript and figures.

When describing the nature of the PhDEF/phdef alleles in the different mutants the authors first write PhDEF+6 to describe the phdef allele from which the transposon excised with an in-frame footprint. In lines 269-272 however, they write 'In summary, ...or an in frame PhDEF allele..... This suggested.......was carrying a functional PhDEF allele. I was at first a bit confused by that, assuming that 'functional PhDEF allele referred to the WT allele'. This can be easily solved by writing 'functional PhDEF+6 allele'.

The term "functional *PhDEF* allele" was meant to include both wild-type *PhDEF* allele and *PhDEF+6* alleles. Indeed, both alleles have been found in star flowers, (see Fig. 2C) but the analysis of the star progeny was only conducted on a flower carrying a *PhDEF+6* allele. We have added that "functional *PhDEF* allele" referred to either a wild-type *PhDEF* or *PhDEF+6* allele, lines 267-268 of the revised manuscript.

- First, the authors conclude in the results that the milder effect in the wico petal tube and star petal limb compared to the full phdef knock-out is due to cell non-autonomous effects, with the PhDEF+6 functional allele in the L1 (wico) and L2 (star) layer partially rescuing the mutant phenotype. When reading 'cell non-autonomous effect', I assumed that the authors meant transport of the mRNA or protein between cells. In the Results section, it remains vague what kind of 'cell non-autonomous effects' the authors think of. In the Discussion section, they elaborate much better on it. The mRNA is not mobile (see in situ's), so it should be the protein that is transported. This is not so likely however, given for example the non-pigmented limb in the star mutant. The influence of mechanical forces from the neighbouring 'wild-type' tissue seems more likely. Only in the Discussion section, the authors explain that they tried to test this, but didn't manage and there, they also elaborate about possible mechanical forces. I think that it would be better if the authors would already mention this earlier (in the Results section), to prevent confusion of the reader. The discussion part about the non-autonomous effects is perfect, but explaining it already at an earlier stage the different kind of non-autonomous effects that are considered would make it clearer. I'm also not sure whether mechanical forces can be considered as cell non-autonomous effects. Because the petal limb cells in star are never pigmented, I think that two explanations are plausible:

o Protein movement only at a very early stage of petal development, in the initiating primordium. This would explain why cell form/shape may be partially rescued, but petal pigmentation not.

o Mechanical effects of the neighbouring cells (most likely).

We agree that the possible nature of the non-autonomous effects appeared very vague at the first reading of the Results section. We have now added two sentences (lines 327-332) explaining that these effects might be mediated by molecular movement (PhDEF or another molecule) and/or by mechanical effects, which we discuss in larger details in the Discussion. To our opinion, mechanical effects can be considered as mediators of non-cell-autonomy, since it is a

physical mean by which a cell with a given genotype acquires features from another genotype, but we agree that it is not the most obvious mechanism that readers will have in mind. We have also modified the paragraph of the Discussion about non-cell-autonomy, to integrate some insights from the reviewer.

The RNA-seq indicates that PhDEF regulates several genes from the anthocyanin pathway. The authors check whether the main players in the pathway have MADS-domain binding sites in their regulatory region (CArG-boxes), indicating that those may be directly regulated by PhDEF. However, to search for CArG-boxes, the authors have invented a complicated method that is not entirely correct. The JASPAR TF motif database contains PWM matrices for about 20 different MADS-domain proteins, which all come down to the canonical CC[A/T6]GG motif or the C[A/T8]G alternative. In my experience, searching regulatory regions fort he presence of these motifs, using a reasonable JASPAR threshold score of 0.90 - 0.95%, will yield about 1 motif per gene. However, the authors find many more per gene, indicating that they put the JASPAR threshold too low (indeed, they put it at 0.86). In this case, also motifs with for example a C in the middle of the A/T tract will be identified (while these are never bound). Subsequently, the authors identify 'high-confidence binding sites' as those sites bound by multiple MADS-box proteins (i.e. based on the JASPAR PWMs), but with the used threshold, this may still be a false positive hit. A better approach would be: screen with all MADS PWMs simultaneously at a higher threshold and check by eye wether the identified CArG-box is a good one.

- Figure 6C-E can then be removed, and replaced by panels that show in addition to the position of the CArG-box in the regulatory region, also the sequence of the identified CArG-boxes.
- Lines 413 - 426 can then simply be replaced by 'the regulatory regions of AN1, AN2 (and PhDEF) were screened for the presence of CArG-boxes (MADS-domain TF binding sites) using the JASPAR database.

Thank you for your input on predicting CArG boxes with JASPAR. We had chosen a low score threshold (0.86) on purpose, in order to have a view of the background of predicted sites, and better detect the most likely CArG boxes. We understand that this might have rendered Figures 6C-E difficult to read, and gave the impression that way too many CArG boxes were present in the sequences analyzed. We are well aware that all the dots in Figure 6 are not CArG boxes, and the most likely ones are probably above the score of 0.9. The term "high-confidence CArG boxes" probably induced some confusion, giving the impression that we believed other dots on the figure were also CArG boxes, but low-confidence ones. We have now replaced the term "high-confidence CArG boxes" by "putative CArG boxes". Moreover, to improve readability of the figures, we have now put the threshold at 0.9.

The predictive score is a quantitative view of the in vitro binding of a MADS protein to a motif, and scores are very dependent on the length of the motif (which varies between 10 and 20 nucleotides- long in the 24 Jaspar MADS motifs that we used); therefore we reasoned that putting an absolute threshold could be too restrictive. Moreover, a consensus sequence only has a low predictive power to detect CArG boxes (for SEP3, only 17% of actual binding sites contain a consensus CArG box, from Kaufmann et al. 2009) and position weight matrices, although not perfect, integrate much more information about the binding preferences than a consensus would. Still, we agree that looking at the individual sequence of the predicted CArG box is a necessary step to check that putative sites match what is known about MADS-box TFs binding.

We have looked at the sequence of the CArG boxes that we identified: *AN2-bs3* is a canonical CArG box with the sequence CCATAATAGG. *AN1-bs1* is also a canonical CArG box with the sequence CTATATTTGG.

We have now added this information in the Results section, lines 426-428 of the revised manuscript.

- It is a bit unexpected that the experimentally tested CArG-boxes are in the terminator regions of AN1 and AN2. Enhancer elements can be located at the 3'end, but are then usually combined with a motif at the 5'end of the CDS. In this case, a loop can be formed for very efficient expression. In particular for B-class MADSes, which are thought to form a tetrameric complex, a double functional CArG-box would make sense. I suggest that the authors redo their JASPAR analysis and test additional (canonical) CArG-boxes at the 5' site, if these exist. Possibly, a good site can then also be identified in AN1.

We fully agree that the fact that the two most promising CArG boxes that we found in AN1 and AN2 are in the terminator region is surprising.

For *AN2*, as shown in Fig. 6E, if putting the score threshold for CArG box prediction at 0.9, there is one putative CArG box (that we called *AN2-bs1*) at position 2135 (i.e. 866 bp upstream the START codon) with the non-canonical sequence GAAAAGTAG. We have tested this element by gel-shift assay and, as could be expected from the presence of a G in the middle of the AT-tract of the CArG box, we could not detect any binding of PhDEF+PhGLO1. There is no additional element worth testing in the promoter, therefore there is likely no DNA-looping between the promoter and terminator mediated by MADS-box proteins only. Still, it is possible that other interactors of PhDEF (since MADS-box proteins interact with very different proteins, see for instance van Mourik et al. 2023 for FUL interactors) might mediate this terminator-promoter looping. We have also tested the site *AN2-bs2*, located in the terminator region of *AN2* but much closer to the STOP codon than *AN2-bs3*, with the idea that with two close binding sites, a MADS quartet might form in the terminator region of *AN2*. However this site is clearly not a canonical CArG box (TCTTTTTTAA) and was not bound in our gel-shift assay. We have added these pieces of information in the Discussion section (lines 621-627), and added the gel shift assay for *AN2-bs1* and *AN2-bs2* in Supplemental Figure 8, together with elements about *AN2-bs3* conservation as requested by Reviewer 1.

For *AN1*, as shown in Fig. 6D, there is really no element in the promoter that is worth testing by gel-shift assay. However, there are putative CArG boxes in the longest intron of the gene that might be bound by PhDEF, which we have not tested. Our goal in this manuscript was not necessarily to identify all possible PhDEF binding sites in *AN1* and *AN2*, and we decided to focus on the most promising one for each gene, based on the CArG box predictions. We agree that the mechanism of action of PhDEF and its protein partners to potentially activate *AN2* expression is intriguing and would deserve further investigation, but to our opinion this represents a whole other study that is beyond the scope of our manuscript.

- The authors write in lines 455-456: 'no strong enrichment was detected in any of the AN1 genomic fragments'. However, though quite mild, there is enrichment for AN1-bs2 (unexpected given the EMSA results), and I think that the authors should mention this.

Indeed, we found a weak but significant enrichment for *AN1*^{GF2} (which is located in the 7th exon of the *AN1* gene, but it does not correspond to the position of *AN1-bs2* that was not bound in the EMSA). Since there is no putative CArG box in this region, we think that we might have identified by chance the indirect binding of PhDEF to this region, mediated by the direct binding of another transcription factor in complex with PhDEF. However, since the enrichment is extremely weak, this region does not match a predicted CArG box and was found purely by chance, we prefer not to highlight it in our manuscript since it would point *AN1* as a binding target of PhDEF for dubious reasons. We have therefore decided not to modify this part of the manuscript.

- There are several small spelling errors or typos:

o Line 170, simultaneous

o Line 292, limb should be limbs

o Line 298, than should be as

o Line 523, restaures should b erestores

o Lines 530+534, 'participate to' should be 'participate in' (or contribute to)

o Line 615, 'our data indicates' should be 'our data indicate'

Thank you for noticing these typos, we have corrected all of them.

TPC2023-RA-00287R2 2nd Editorial decision – *acceptance pending* **September 17, 2023**

We are pleased to inform you that your paper entitled "Cell layer-specific expression of the homeotic MADS-box transcription factor PhDEF contributes to modular petunia petal morphogenesis" has been accepted for publication in The Plant Cell, pending a final editorial review by a science editor. At this stage, your manuscript will be evaluated by a science editor with respect to its presentation of scientific content, compliance with journal policies, and presentation for a broad readership.

Final acceptance from Science Editor **October 3, 2023**
